# Targeting Mcl-1 by a small molecule NSC260594 for triple-negative breast cancer therapy

**DOI:** 10.1038/s41598-023-37058-4

**Published:** 2023-07-22

**Authors:** Shengli Dong, Margarite D. Matossian, Hassan Yousefi, Maninder Khosla, Bridgette M. Collins-Burow, Matthew E. Burow, Suresh K. Alahari

**Affiliations:** 1 TYK Medicines Inc., Block D, No. 778 Huaxi Avenue, Changxing, 313100 Zhejiang People’s Republic of China; 2 Department of Biochemistry and Molecular Biology, LSUHSC, CSRB 406, New Orleans, LA 70112 USA; 3 Stanley S. Scott Cancer Center, LSUHSC School of Medicine, New Orleans, LA 70112 USA; 4 Tulane University School of Medicine, New Orleans, LA 70118 USA

**Keywords:** Breast cancer, Cancer therapy, Biochemistry, Cancer

## Abstract

Triple-negative breast cancers (TNBCs) are aggressive forms of breast cancer and tend to grow and spread more quickly than most other types of breast cancer. TNBCs can neither be targeted by hormonal therapies nor the antibody trastuzumab that targets the HER2 protein. There are urgent unmet medical needs to develop targeted drugs for TNBCs. We identified a small molecule NSC260594 from the NCI diversity set IV compound library. NSC260594 exhibited dramatic cytotoxicity in multiple TNBCs in a dose-and time-dependent manner. NSC260594 inhibited the Myeloid cell leukemia-1 (Mcl-1) expression through downregulation of Wnt signaling proteins. Consistent with this, NSC260594 treatment increased apoptosis, which was confirmed by using an Annexin-V/PI assay. Interestingly, NSC260594 treatment reduced the cancer stem cell (CSC) population in TNBCs. To make NSC260594 more clinically relevant, we treated NSC260594 with TNBC cell derived xenograft (CDX) mouse model, and with patient-derived xenograft (PDX) organoids. NSC260594 significantly suppressed MDA-MB-231 tumor growth in vivo, and furthermore, the combination treatment of NSC260594 and everolimus acted synergistically to decrease growth of TNBC PDX organoids. Together, we found that NSC260594 might serve as a lead compound for triple-negative breast cancer therapy through targeting Mcl-1.

## Introduction

Breast cancer has surpassed lung cancer and become the most common diagnosed cancer overall in 2020^1^. Although considerable progress has made in the treatment of human primary breast cancer in past decades, breast cancer is still the second leading cause of deaths among American women. In 2021, there were 284,200 new breast cancer cases and about 44,130 patients who died from breast cancer in the United States^2^. TNBCs, which account for 10–20% of all breast cancer patients, are aggressive forms of breast cancer and tend to grow and spread more quickly than most other types of breast cancer^3^. TNBCs have relatively high rates of recurrence and distant metastasis, and patients have overall poor survival^3^. TNBCs do not express estrogen (ER), progesterone (PR) receptors and the HER2 protein on the cell surface, which is the reason for the name “triple negative”, therefore they cannot be cured by hormonal therapies such as tamoxifen or aromatase inhibitors. Similarly, they do not respond to immunotherapies that target the HER2 protein, for example through the monoclonal antibody trastuzumab. Therefore, there are urgent unmet medical needs to develop targeted drugs for TNBCs.

What makes cancer cells different from normal cells is that cancer cells can escape from apoptosis, thus allowing them to grow almost indefinitely^4^. Apoptosis is the programmed cell death that occurs as the normal and controlled part of an organism’s growth. Apoptosis is tightly regulated by the B-cell lymphoma-2 (BCL-2) family proteins, which include both pro-apoptotic and anti-apoptotic proteins. A delicate balance in the relative ratio of anti-apoptotic to pro-apoptotic proteins is necessary for the regulation of cell survival. Cancer cells often upregulate anti-apoptotic proteins to evade apoptosis^5–7^. One strategy to kill cancer cells is to inhibit the overexpressed anti-apoptotic proteins so that apoptosis can take place in these cells. Anti‐apoptotic BCL-2 family proteins include Bcl-2, Bcl-xL, Bcl-w and Mcl-1. Many studies suggest that small-molecule inhibitors of pro‐apoptotic BCL-2 family proteins are very promising in cancer therapy^5,8^. For example, venetoclax (ABT-199), a selective Bcl-2 inhibitor, has been approved by FDA for clinical use in chronic lymphocytic leukemia in 2016^9^.

In this study, we identified a small molecule NSC260594 from the National Cancer Institute (NCI) diversity set IV compound library, which efficiently inhibited growth of TNBC cells. In addition, we identified Mcl-1 as the target of NSC260594 in TNBC cells and demonstrated that NSC260594 inhibited Mcl-1 expression through downregulation of Wnt signaling proteins. The MCL1 gene is the most frequently amplified gene (following TP53) after neoadjuvant therapy in TNBC and this is associated with a unfavorable outcome for the patients^8,10^. Moreover, Mcl-1 is a pro-survival protein that has been implicated in the maintenance of chemotherapy-resistant cancer stem cells (CSCs) in TNBC^11^. In many cases, cancer cell resistance can be countered through treatments that decrease, weaken or turn off MCL-1^12–14^. As a result, MCL-1 has become a promising target for cancer therapy. We developed a novel combination therapeutic strategy for TNBC tumors in this study.

## Results

### NSC260594 efficiently killed TNBC cells in a dose- and time-dependent manner

**Figure 1:**
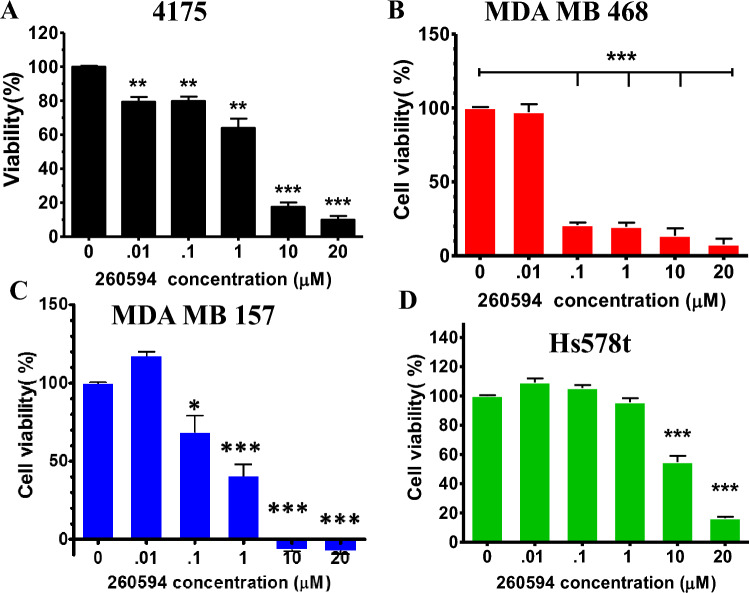
NSC260594 treatment efficiently killed multiple TNBC cell lines in a dose-dependent manner. We treated (**A**) 4175 cells; (**B**) MDA-MB 468 cells; (**C**) MDA-MB 157 cells; and (**D**) Hs578t TNBC cells with different dosage of NSC260594 for 72 h. Cell viability was determined in quadruplicate using the MTT method. NSC260594 treatment efficiently killed heterogeneous TNBC cell lines in a dose-dependent manner. Data shown represent the mean ± SD from three independent experiments, each performed in triplicate. Statistically significant values of *p < 0.05, **p < 0.01, and ***p < 0.001 were determined compared with the control.

In order to examine the capability of inducing TNBC cell death with the small molecules from the NCI diversity set IV compound library, we treated the most commonly used human TNBC cell line MDA-MB 231 with the library compounds and found that NSC260594 treatment efficeintly killed MDA-MB 231 cells in a dose- and time-dependent manner (SFig. 1A and 1B). The calculated IC_50_ of NSC260594 was 4 μM for MDA-MB 231 cells (SFig. 1C). This value falls in a favourable drug usage range. TNBC is a highly heterogeneous cancer. To exclude the possibilty of NSC260594 working only on a single cell line, we treated four more human TNBC cell lines with NSC260594. As shown in Fig. 1 and SFig. 2, NSC260594 treatment efficiently killed 4175 (IC_50_ = 0.85 μM) , MDA-MB 468 (IC50 = 0.31 μM) , MDA-MB 157 (IC_50_ = 1.94 μM) , SUM159PE (IC50 = 0.95 μM) and Hs578t cells (IC50 = 4.48 μM) , but not the mouse embryonic fibroblast (MEF) cells (Fig. 1 and SFig. 3, data not shown) .

**Figure 2:**
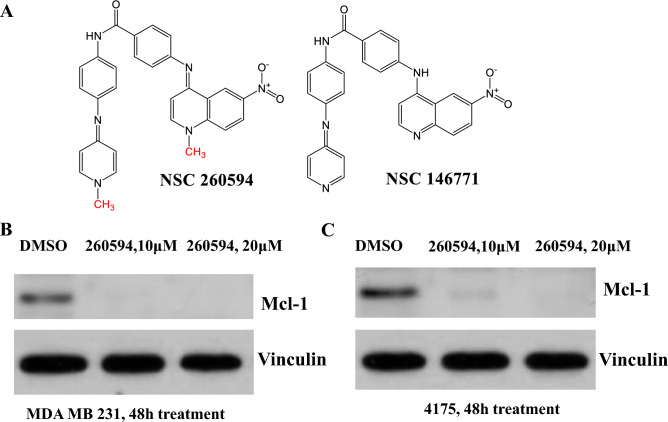
Identification of NSC260594 as a Mcl-1 inhibitor. (**A**) Structure of NSC260594 (260594) and NSC146771. (**B**–**C**) experiments demonstrating 260594 is a Mcl-1 inhibitor. MDA-MB231 cells (**B**) and 4175 cells (**C**) were treated with 260594 or DMSO as control for 48 h. The protein level of Mcl-1 was examined by Western blot. Vinculin was used as a loading control. 260594 significantly inhibited Mcl-1 expression in both TNBC cell lines.

Next we examined the effect of NSC260594 on inhibiting Mcl-1 protein expression. Mcl-1 is an anti-apoptotic protein; high Mcl-1 protein expression often predicts poor prognosis in TNBCs^8^. Using a competitive fluorescence polarization assay and computational method, a UK group found that small molecule NSC146771 was a selective Mcl-1 inhibitor in pancreatic cancer cells^15^. NSC260594 is a quinolinium derivative and an analog of NSC146771. Since the stereo structure of NSC260594 is very similar to NSC146771 (Fig. 2A), we hypothesized that NSC260594 might inhibit Mcl-1 in TNBCs. Thus, we treated human TNBC MDA-MB 231 and its more aggressive derivative 4175 cells, with NSC260594 for 48 h, and found that NSC260594 completely inhibited Mcl-1 expression in both cell lines (Fig. 2B). We conclude that NSC260594 is an inhibitor of Mcl-1 and it may be a viable drug to treat TNBCs.

### NSC260594 inhibits Mcl-1 expression through downregulation of Wnt signaling proteins

**Figure 3:**
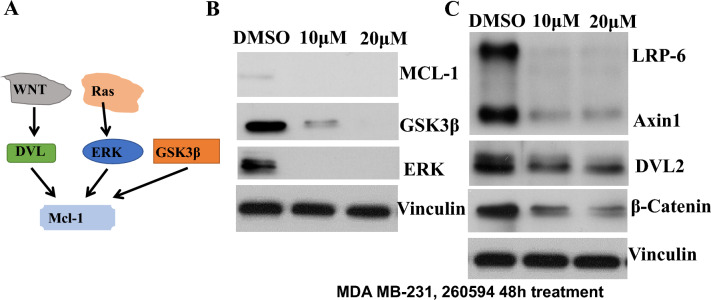
Delineation of molecular mechanisms underlying NSC260594 effects on Mcl-1 inhibition and cancer cell killing. (**A**) The possible pathways regulate Mcl-1. (**B**) 260594 treatment inhibited GSK-3β, and ERK. (**C**) 260594 treatment inhibited the key players of Wnt signaling pathway.

We further attempted to delineate the molecular mechanism underlying NSC260594 action on Mcl-1 inhibition. Aberrant activation of Wnt signaling pathway contributes to tumorigenesis of TNBCs^16^. β-catenin and GSK-3β expression are key players of Wnt signaling pathway. β-catenin and GSK-3β modulate apoptosis by destabilization of Mcl-1 through c-Myc in TNBCs. C-Myc is a Wnt targeting gene. Mcl-1 and c-Myc are frequently co-amplified in TNBCs^17^. We found that NSC260594 treatment significantly suppressed the protein levels of the major players including Dvl2, axin1, LRP6, β-catenin and GSK-3β in Wnt signaling pathway in Fig. 3B,C. In addition, previous studies revealed that ERK phosphorylated Mcl-1 and up-regulated Mcl-1 expression{Dong, 2008 #38}. We found that NSC260594 treatment inhibited Erk1/2 (Fig. 3 and SFig.4) in MDA-MB-231 cells. Together, our data suggest that NSC260594 treatment inhibits Mcl-1 expression through downregulation of Wnt signaling pathways in TNBCs (Fig. 3A).

### NSC260594 treatment reduces drug-resistant cancer stem cell (CSC) population in TNBCs

**Figure 4:**
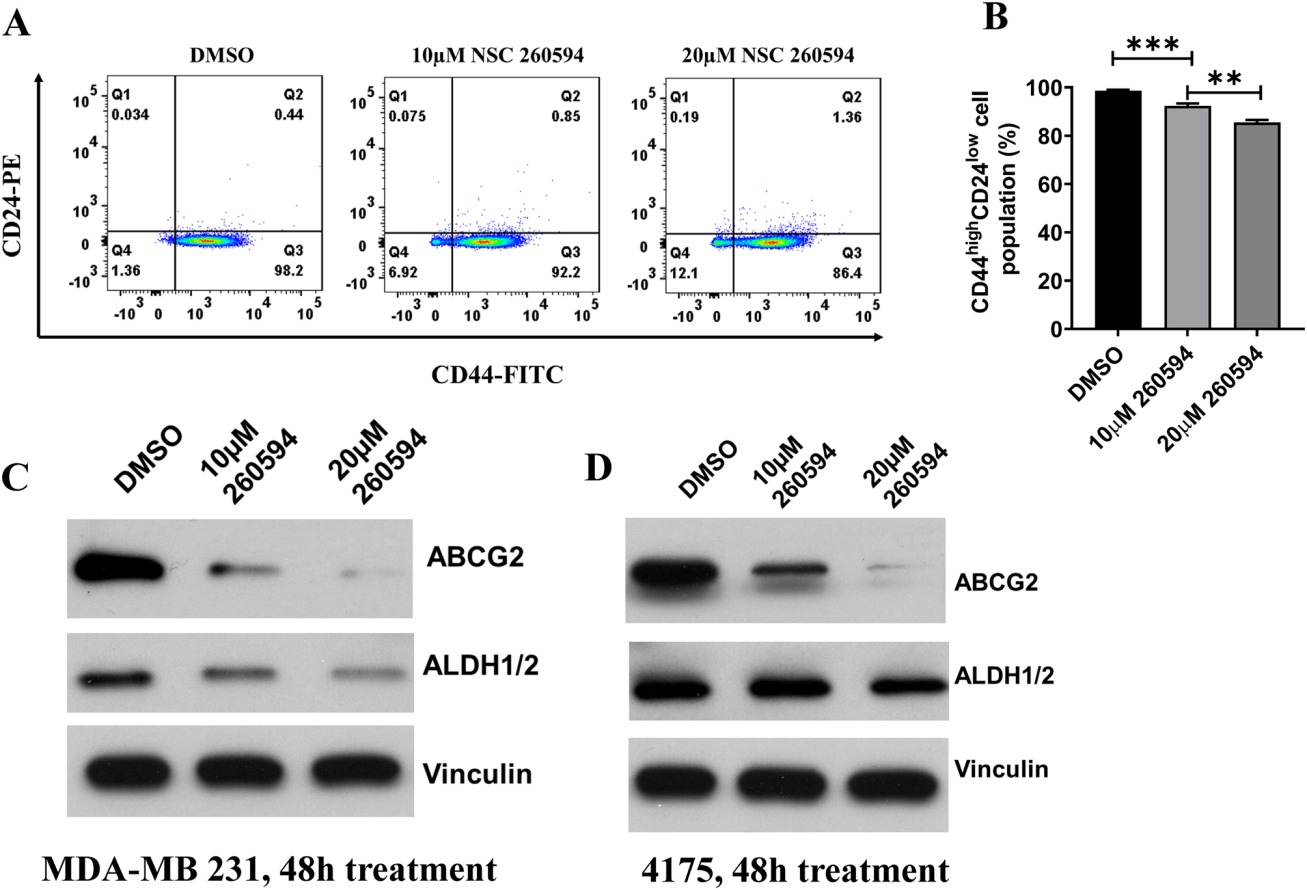
NSC260594 treatment reduces cancer stem cell (CSC) population in TNBCs. (**A**) MDA-MB231 Cells were treated with NSC260594 and detached with versene/0.48 mM EDTA. Cells were co-stained by fluorochrome-conjugated antibodies against CD44 (FITC) and CD24 (PE) at 4 °C in the dark for 20 min. The labelled cells were analyzed on a FACS Calibur, the results were analyzed by FlowJo software. (**B**) The population of CD44^high^CD24^low^ cells in A was quantified. (**C**) MDA-MB 231 and (**D**) 4175 were treated with NSC260594 for 48 h, stem cell marker ALDH1/2 and ABC transporters ABCG2 were examined by western blot assay, vinculin was used as protein loading control. Data shown represent the mean ± SD from three independent experiments, each performed in triplicate. Statistically significant values of *p < 0.05, and **p < 0.01 were determined compared with the control.

Mcl-1 is also an important factor in drug resistance. Drug-resistant cancer cells are more tumorigenic than their parental cells. The existence of drug-resistant cancer stem cells is a major obstacle in effectively treating and curing cancers. Drug resistance often results in cancer relapse^18^. Aberrant activation of Wnt signaling has been associated with breast cancer stem cells. Thus, we hypothesized that NSC260594 treatment could overcome drug resistance not only through Mcl-1 inhibition but also through cancer stem cells suppression. Subpopulations of CD44^high^ and CD24^low^ cells^19^, have been shown to function similar to CSCs. Thus, we used MDA-MB-231 cells to evaluate the drugs’ effect on CSCs. After treating these cells with Dimethyl sulfoxide (DMSO) (control) and NSC260594, we examined the number of CD44^high^ and CD24^low^ cells by flow cytometry analysis. Indeed, NSC260594 treatment decreased the population of CD44^high^ and CD24^low^ cells (Fig. 4A,B). In addition, we also found that NSC260594 treatment significantly decreased the expression of cancer stem cell maker ALDH1/2 (Fig. 4C,D). Furthermore, multiple ABC transporters including ABCG2 enhance drug resistance in breast cancer^20^. We found that NSC260594 inhibited the expression of ABCG2 transporter in MDA-MB 231 and 4175 cells (Fig. 4C,D). Together, these data indicate that NSC260594 inhibits cancer stem cell population in TNBCs and this may help overcome the drug-resistance of TNBCs.

### NSC260594 treatment increases the apoptosis in TNBCs

**Figure 5:**
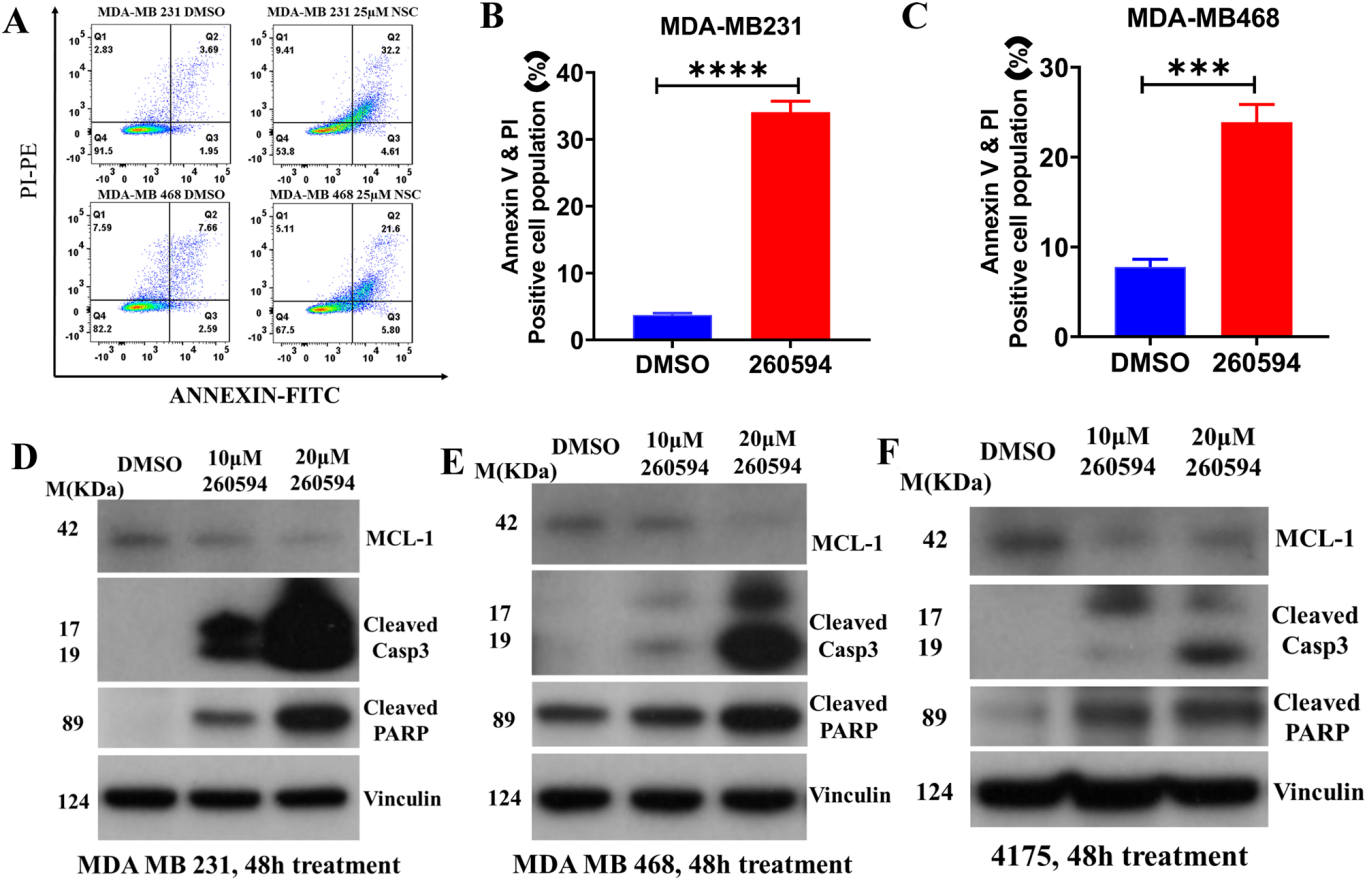
NSC260594 treatment increases the apoptosis in TNBCs. (**A**). Cell apoptosis was detected using an Alexa Fluor® 488 Annexin V/Dead Cell Apoptosis Kit. The cells were processed according to the manufacturer's instructions using a FACS Calibur instrument. The Annexin V and PI positive cells of (**B**) MDA-MB-231 (**C**) MDA-MB468 and (**D**) MDA-MB-468 were counted using FlowJo software. NSC260594 treatment increased the apoptosis markers in MDA-MB-231, (**E**) MDA-MB-468, and (**F**) 4175 cells. Cells were grown in DMEM with 10% FBS overnight. The cells were then treated with DMSO, 10 μM, and 20 μM NSC260594 for 48 h. The proteins including Mcl-1, cleaved caspase 3, and cleaved PARP were detected by western blot assay. Cell cytoskeleton protein Vinculin was used as a protein loading control. Data shown represent the mean ± SD from three independent experiments, each performed in triplicate. Statistically significant values of ***p < 0.001 were determined compared with the control.

Apoptosis is tightly regulated by both pro-apoptotic and anti-apoptotic proteins. We hypothesized that inhibition of anti-apoptotic protein Mcl-1 by NSC260594 may promote apoptosis. Indeed, apoptosis is induced by NSC260594 in MDA-MB 231 and MDA-MB 468 cells as demonstrated by an Annexin-V/PI assay (Fig. 5A). Caspase 3 is an important mediator of apoptosis, and its activation is a key event of apoptosis. Caspase-3 activation results in proteolytic processing of its inactive zymogen into activated 17 KDa and 12 KDa fragments. The cleavage of poly (ADP-ribose) polymerase (PARP) is a well-known biomarker of cells undergoing apoptosis. The activated caspase-3 mediates PARP cleavage. We found that NSC260594 treatment decreased the expression of Mcl-1, and NSC260594 treatment increased the cleavage of both caspase-3 (17/19 KDa) and PARP proteins in MDA-MB 231, MDA-MB 468 and 4175 cells (Fig. 5B–F).

### The combination treatment of NSC260594 and everolimus acted synergistically to kill TNBC

**Figure 6:**
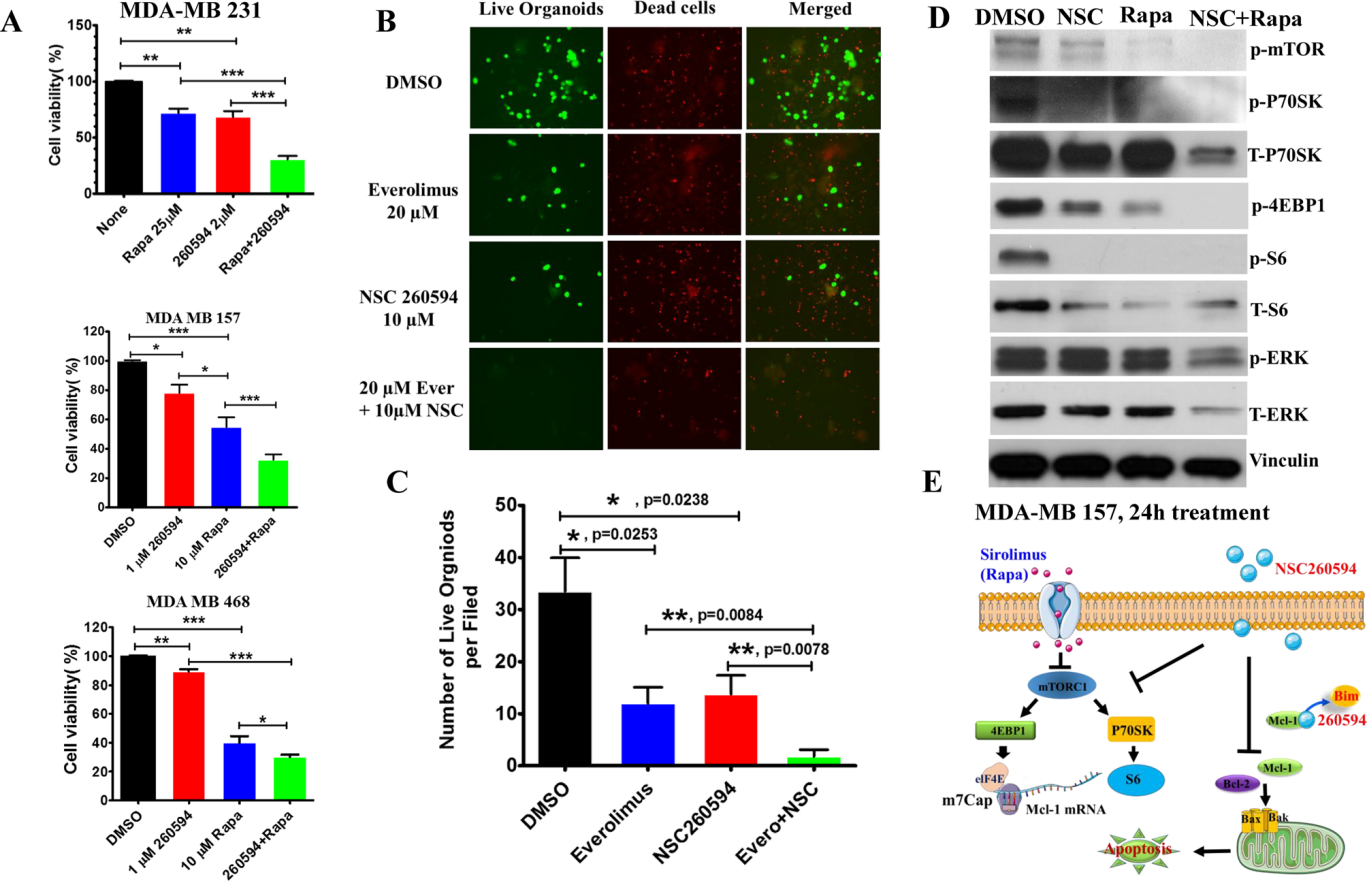
The combination treatment of NSC260594 and everolimus acted synergistically to kill TNBCs. (**A**) NSC260594 and everolimus (Rapa) synergistically killed MDA-MB 231, MDA-MB157 and MDA-MB468 cells. (**B**) NSC260594 and everolimus (Rapa) acted synergistically to kill TU-BcX-4QATb2 3D organoids. PDX-Os were formed from the digested PDX tumor tissue, and then 2000 cells per well were plated in a low attachment 24 well plate. After TU-BcX-4QATb2 organoids were treated with DMSO or NSC260594 (10 μM), Everolimus (20 μM) and 10 μM NSC260594 + 20 μM Everolimus for 72 h, cells were stained with Calcein-AM (2 μM) and Ethidium homodimer (EthD)-III (5 μM) for 45 min using the PromoKine live/dead staining kit. Organoids were imaged with fluorescence microscopy. The 588 nM excitation channel was used to identify red, ‘dead’ cells, and the 420 nM excitation channel was used to visualize green, ‘live’ cells. (**C**) The number of live organoids per filed was quantified. (**D**) MDA-MB 157 cells were treated with DMSO (control), NSC260594, everolimus, and the combination of NSC260594 and everolimus. Expression of mTOR cascade proteins in cell lysates was analyzed by western blot assays. (**E**) The diagram of the potential molecular mechanisms underlying the synergistic effect of NSC260594 and everolimus in killing TNBC cells.

AKT/mTORC1 activation is one of the most commonly deregulated pathways in TNBCs^21^, and Mcl-1 expression is regulated by mTORC1^22^. The mTORC1 inhibitor everolimus, also known as rapamycin (Rapa), is an FDA approved oncologic drug. Mcl-1 overexpression in TNBCs could counteract everolimus-induced apoptosis, causing drug resistance to everolimus. On this basis, we hypothesized that inhibiting Mcl-1 by NSC260594 could improve everolimus-induced apoptosis in TNBCs, and the combination of NSC260594 and everolimus could provide synergistic effects in tumor-killing activity. We treated MDA-MB 231, MDA-MB157 and MDA-MB468 cells with DMSO (control), NSC260594, everolimus, and the combination of NSC260594 and everolimus. We found that the combination of NSC260594 and everolimus acted synergistically to kill MDA-MB 231, MDA-MB157 and MDA-MB468 cells (Fig. 6A). To make combination treatment more clinically relevant, we made patient-derived organoids which were generated from Tu-BcX- 4QATb2 TNBC PDX models. After treating the 3D organoids with DMSO (control), 10 μM NSC260594, 20 μM everolimus, and the combination of 10 μM NSC260594 and 20 μM everolimus for 72 h, the wells were subsequently stained with Calcein-AM to highlight live cells (green) or EthD-III to highlight dead cells (red). NSC260594 and everolimus significantly suppressed the growth of Tu-BcX- 4QATb2 TNBC 3D organoids, and the combination of NSC260594 and everolimus acted synergistically to kill TNBC 3D organoids (Fig. 6B,C). We then aimed to delineate the molecular mechanisms (Fig. 6E) underlying the synergistic effect of NSC260594 and everolimus. Cell lysates were prepared from MDA-MB 157 cells that are treated with DMSO (control), NSC260594, everolimus, and the combination of NSC260594 and everolimus. Expression of mTOR cascade proteins was analyzed by western blot assays. The combination treatment significantly blocked the protein expression of Mcl-1 and p-4EBP1 (Fig. 6D).

### Antitumor activity of NSC260594 in a nude mouse model

**Figure 7:**
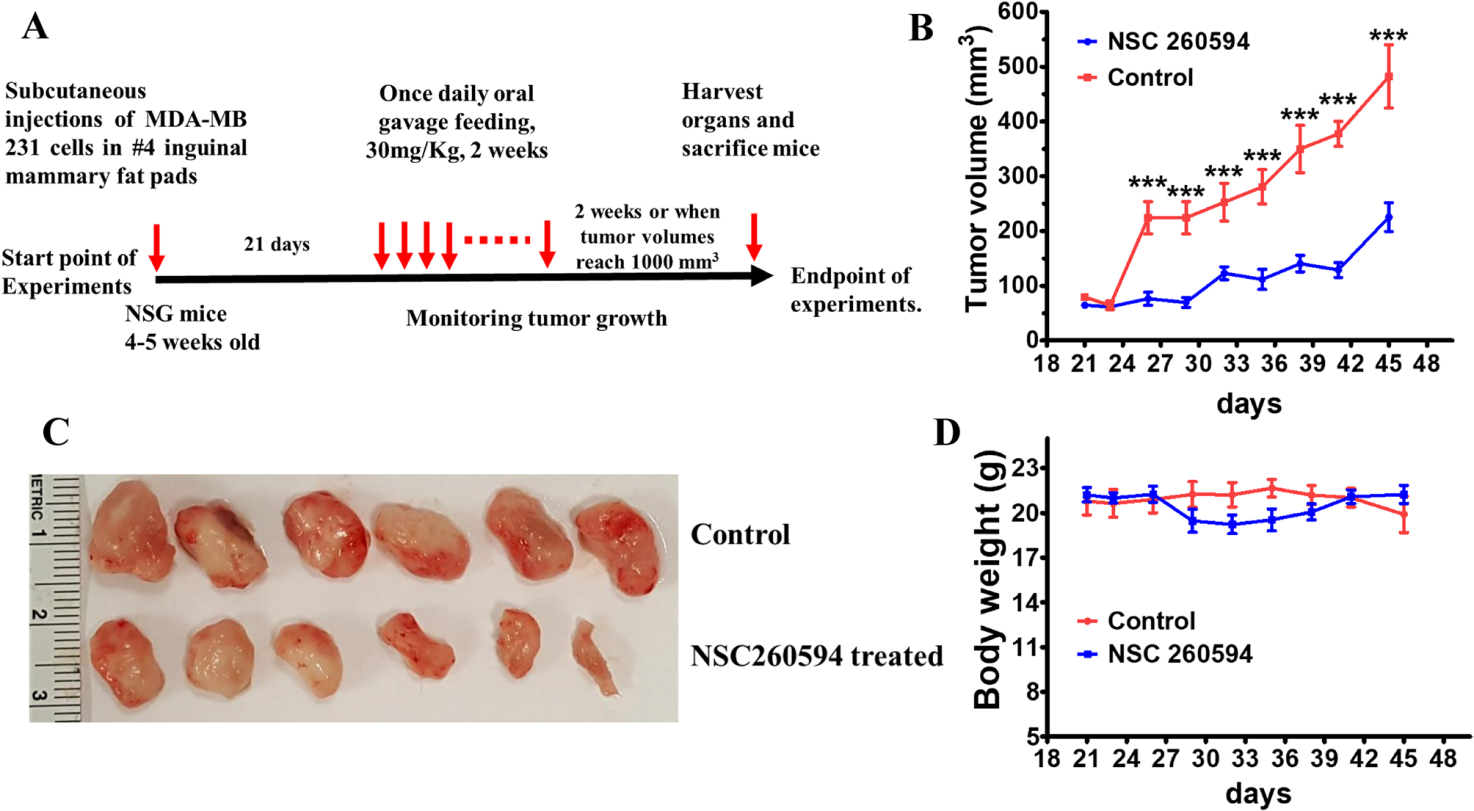
Examine antitumor activity of NSC260594 in a nude mouse model. (**A**) The diagram of animal experimental design. MDA-MB 231 cells with 50% Matrigel were implanted into Number 4 mammary gland of 4–5 weeks old female NSG mice. When tumors reached a size of ∼100 mm^3^, the mice were randomly assigned into two groups: untreated control and NSC260594 (30 mg/kg bodyweight). NSC260594 was administrated by oral gavage once daily for two weeks. (**B**), tumor volume measured over a period of 24 days. (**C**) Size of tumors (**D**) the body weight measured over a period of 24 days. Data shown represent the mean ± SD from three independent experiments, each performed in triplicate. Statistically significant values of *p < 0.05, **p < 0.01, and ***p < 0.001 were determined compared with the control.

MDA-MB-231 cells were implanted in female NSG mice (Jackson Laboratory) by subcutaneous (s.c) injections as described previously^23^. When the tumor volume reaches 100 mm^3^, the mice were treated daily by gavage feeding at 30 mg/kg bodyweight of NSC260594 for two weeks and then watched for other two weeks. Plain sesame oil was used as control (Fig. 7A). NSC260594 treatment significantly suppressed the growth of MDA-MB 231 tumors (Fig. 7B,C). Concordant with these data, we noticed inhibition of cell proliferation as demonstrated by Ki-67 staining and vasculature by CD31 staining (SFig. 5 The bodyweight of mice maintained stable in NSC260594 treatment group (Fig. 7D).

### Antimetastastic activity of NSC260594

We further examined lungs of these mice and found that the drug inhibited metastasis (SFig. 6) suggesting that this compound inhibits metastasis as well.

## Discussion

TNBC is the most aggressive subtype of breast cancer. Overall, its five-year survival is much lower than other subtypes of breast cancer. There has been impressive progress in TNBC targeted therapy in recent years. TNBC tumors carrying BRCA mutations are sensitive to poly-ADP-ribose polymerase (PARP) inhibitor therapy^24,25^. Recently, Olaparib, a PARP1 inhibitor, received FDA approval to treat advanced-stage TNBC with a BRCA1 or BRCA2 mutation^26,27^. PD-L1 is expressed in about 40% of TNBC tumors, adding an anti-PD-L1 antibody atezolizumab to nab-paclitaxel chemotherapy significantly improved PD-L1 positive TNBC survival. Despite the successes of PD-L1/PD-1 inhibitors, PARP inhibitors, and anti-Trop-2 antibody drug conjugates in TNBC, 60% of TNBC patients’ tumors do not express PD-L1 and only about 20% of TNBC patients carry germline BRCA1/2 mutations^28^. As a result, many TNBC patients would not benefit from these recently FDA-approved targeted therapies because of no biomarkers. Therefore, there are urgent needs to develop new strategies for TNBC treatment.

In this study, we identified a small molecule NSC260594 as a potential therapeutic agent from the TNBC drug screening of the NCI diversity set II compound library. The IC50 of NSC260594 for TNBC cells in a range between 0.5 and 10 μM SFig.1, and SFig. 3). It suggests this compound is a good lead compound for further development. Little is known about the function of NSC260594. PubMed search shows only a total of six publications related to this compound, and none of them examined its effects on cancer cells. Two of these studies indicate that NSC260594 may have antiviral and anti-parasitic activity^29,30^. Through a polarization assay, Howell LA group found NSC146771 was a Mcl-1 binder. Computational docking experiments demonstrated NSC146771 is a BH3 mimetic, which binds in the mNoxa binding groove^15^. NSC260594 is an analog of 146771. Therefore, NSC260594 could bind the shallow groove of the Mcl-1 protein and inhibit the Mcl-1 activity. Pro-apoptotic protein Bax and Bak are “effectors”. Bak/Bax oligomerizes by binding to “activators” (Bim, Bid, and Puma) at the outer mitochondrial membrane, resulting in cytochrome-c release, apoptosome formation and caspase-dependent apoptosis. Anti-apoptotic Bcl-2 family proteins (Bcl-2, Bcl-A1, Bcl-xL, Bcl-w, and Mcl-1) favor cell survival by binding and sequestering effectors and activators. Cancer cells evade apoptosis by increasing levels of anti-apoptotic Bcl-2 proteins including Mcl-1^6,7^. We hypothesize that NSC260594 will inhibit Mcl-1 and induce cancer cell apoptosis in TNBCs.

In addition, NSC260594 modulates Mcl-1 expression not only at the protein level but also at the transcription level. We found that NSC260594 treatment suppressed Mcl-1 expression (Fig. 2). There is a G/C rich area in the promoter region and at the splice sites of human Mcl-1 gene^31^. This G/C rich region will form a DNA or RNA-type secondary structure named G-quadruplex. G-quadruplex is an emerging therapeutic target in oncology since it is possible to suppress oncogenes including Mcl-1 through stabilization of these structures. G-quadruplexes have regulatory roles in human telomeres, oncogene-promoter regions, replication initiation sites, and 5'- and 3'- UTR of mRNA^32^. Using FRET melt, circular dichroism, cytotoxicity MTS assays, and RT-qPCR methods, Jenkins et al. found that NSC260594 could stabilize the G-quadruplexes within the ADAM-15 promoter^33^. Moreover, NSC260594 could recognize G9-G10-A11-G12 RNA tetraloop of HIV and prevent the binding of the Gag protein within the 5′-UTR^29,34^. Together, NSC260594 has the potential to suppress Mcl-1 expression through stabilizing G-quadruplexes. It warrants an investigation whether NSC260594 regulates Mcl-1 gene expression through G-quadruplexes in its promoter region or at the splice sites.

Crosstalk between th PI3K/AKT pathway and other canonical signalling pathways is well-characterized, and these pathways drive tumorgenesis and promote resistance to therapies^35^. Multiple genomic alterations results in PI3K pathway activation, including oncogenes PI3KCA, AKT and MTOR activition or tumour suppressor genes inactivation such as INPP4B, PTEN, TSC1/2 and LKB1. For example, PI3K3CA is the second most frequently mutated gene in TNBC. Together with additional PTEN and AKT1 mutations/alterations, PI3K/AKT pathway activation occurs in about 25% of primary TNBC and possibly a higher frequency in metastatic TNBC. An important consequence of PI3K/AKT pathway activation is mTOR stimulation. mTORC1 activation increases eIF4E, and higher eIF4E levels will further increase the translation of mRNAs having lengthy, G + C-rich, highly structured 5’ UTRs^36,37^. As previously mentioned^31^, Mcl-1gene has a G + C-rich (> 70%) 5’ UTR. Therefore, Mcl-1 expression is regulated by mTORC1 and Mcl-1 mRNA translation is an important target of everolimus(rapamycin)^36^. We found that the combination of NSC260594 and everolimus provided synergistic effects in tumor-killing activity in TNBC. Our findings suggest a novel avenue for TNBC treatment.

## Methods

### Experimental animals and ethical statement

All mice were housed in a pathogen-free animal room under standard conditions with free access to water and food (standard chow diet and water ad libitum). All procedures were approved by the Institutional Animal Care and Use Committee of the LSU Health Science Center at New Orleans. Mice were euthanized using Co2 inhalation. The study is reported in accordance with ARRIVE guidelines. All methods were performed in accordance with relevant guidelines and regulations.

### Antibodies, cell lines and chemicals

MDA-MB-231, MDA-MB-468, Hs578t, and MDA-MB-453 purchased from the American Type Culture Collection (Manassas, VA). TNBC lung metastatic cell line MDA231-LM2-4175 (4175) cells were obtained from Dr. Joan Massague at Memorial Sloan-Kettering Cancer Center. The MDA231-LM2-4175 cells, a subline of MDA-MB-231, is highly metastatic to the lung transduced with HSV1-TK. SUM159PE cells were kindly provided by Dr. Jennifer A. Pietenpol (Vanderbilt-Ingram Cancer Center). Mcl-1, AKT, PARP, ABCG2, ALDH1/2, Myc, GSK3β, β-catenin, DVL2, Axin1, Anti-phospho-AKT (Ser473), anti-phospho-S6K1 (S79), Anti-phospho-4-EBP1, Anti-phospho-LRP6, Anti-phospho-mTOR, anti- ribosomal protein S6, and anti-cleaved Casp3 antibodies were obtained from Cell Signaling Technology (Beverly, MA). Anti-phospho-ribosomal protein S6 (S235/236), mouse anti-ERK antibody and Anti-phospho-ERK were purchased from Santa Cruz Biotechnology (Santa Cruz, CA). CD-24PE and CD44-FITC were purchased from BD Biosciences. Anti-Vinculin was purchased from Sigma. Secondary anti-mouse IgG with horseradish peroxidase was from Calbiochem. Secondary anti-rabbit IgG with horseradish peroxidase was from GE Healthcare. Paclitaxel was purchased from APExBIO. Everolimus (rapamycin) was purchased from Topscience. The NCI diversity set II compound library and NSC260594 were kindly provided by Developmental Therapeutics Program of NCI. The authentication of the cell lines used in this study was confirmed by short tandem repeat (STR) genotyping analysis, mycoplasma detection kit (Thermo Fisher) was regularly performed to test no contamination.

### Phospho-RTK array analysis

To determine which receptor tyrosine kinases (RTKs) are targeted by NSC260594, the Human RTK Phosphorylation Antibody Array Kit (R&D human phosphor-kinase array, Cat No.: ARY003B) was used. MDA-MB-231 cells were grown in DMEM medium containing 10% fetal bovine serum (FBS) and penicillin/streptomycin. When cells reached 70 ~ 80% confluence, media were changed to Dulbecco's Modified Eagle Medium (DMEM) supplemented with DMSO, or 20 µM NSC260694. After 24 h incubation, cell lysates were prepared using lysis buffer containing protease and phosphatase inhibitors. The membrane blots were developed, and images were acquired as previously described. The relative intensities of the duplicated spots were normalized to positive control spots. Values represent the mean of duplicate spots for each protein after normalization.

### Drug treatments for TNBC cells

Tumor cells were seeded onto 6-well plates at a density of 300,000 cells per well. After culturing in complete DMEM medium for 16 h, media was replaced with fresh DMEM containing 10% FBS and 10 µM and 20 µM for 24 or 48 h. In control conditions, media was replaced with fresh DMEM containing 10% FBS. Protein lysates were prepared as previously described. Samples were boiled in SDS sample buffer for 10 min and stored at − 80 °C until analysis.

### Cell viability assay

TNBC cells were seeded (3,000–5,000 cells per well) in 96-well plates. Growth medium was replaced with either fresh medium (DMSO as a control) or medium containing the drugs for 72 h after overnight growth. Cell viability was determined in quadruplicate using the MTT (3-(4,5-dimethylthiazol-2-yl)-2,5-diphenyltetrazolium bromide) assay. The replicates normalized to the control wells. To further confirm the results of MTT assay, the experiments were repeated using Cell Counting Kit 8 (WST-8 / CCK8) (Abcam, ab228554). The data analysis performed using Prism software (GraphPad Software). Data represent the mean ± SEM. Student's t test was used to analyze the data, and a *p*-value of < 0.05 was considered statistically significant. * *P *< 0.05; ** *P *< 0.01, *** *P *< 0.0001.

### Apoptosis assays

Cell apoptosis was performed using an Alexa Fluor®488 Annexin V/Dead Cell Apoptosis Kit (Invitrogen, Carlsbad, CA, USA) according to the manufacture’s protocol. In brief, 5 × 10^5^ cells were exposed to NSC260594 or DMSO control in 6-well plates and incubated for 48 h before analysis. The cells were then harvested and analyzed according to manufacturer’s instructions using a BD FACS Calibur instrument. The cells that were positive for Annexin V alone, and Annexin V & PI were counted using FlowJo software.

### Flow cytometry assay

MDA-MB231 Cells were washed with cold phosphate-buffered saline (PBS) and then harvested with versene/0.48 mM EDTA (Gibco). Detached cells were resuspended in PBS with 0.5% FBS. Combinations of fluorochrome-conjugated antibodies against human CD44 (FITC; cat. #555478) and CD24 (PE; cat. #555428), or the respective isotype controls, which were obtained from BD Biosciences (California, USA), were added to the cell suspension as the manufacturer’s protocol, and incubated at 4 °C in the dark for 20 min. The labeled cells were analyzed on a FACS Calibur (BD Biosciences), the results were analyzed by FlowJo software.

### Analysis of MDA-MB 231 xenograft tumor development

MDA-MB 231 (1 × 10^6^ cells) with 50% Matrigel™ were injected into Number 4 mammary gland of 4–5 weeks old NSG (Jackson Laboratory). Tumors were measured by a caliper. When tumors reached a size of ∼100 mm^3^ the mice were randomly distributed into two groups (ten mice in each group): untreated control and NSC260594 (30 mg/kg bodyweight). NSC260594 was suspended in sesame oil and administrated by oral gavage once daily for two weeks. Tumor volume measured twice a week after the initial injection, and the volumes were calculated using the formula (π x length x width1 x width2 /6). Mice were euthanized when they became moribund, or when they lost 20% weight. All organs examined for the presence of tumors and metastases at autopsy. Data grouped and plotted using GraphPad Prism 8.

### Patient-derived xenografts and Live/dead fluorescent stain for 3D Organoids

Tu-BcX-4QATb2 PDX tumors were established in 4–6 week-old SCID/Beige (CB17.Cg-PrkdcscidLyst bg/Crl) mice provided from Jackson Laboratory. PDX-Os were formed from the digested PDX tumor tissue, and then 2000 cells per well were plated in a low attachment 24 well plate. The methods for generating PDX organoids (PDX-O) have been previously described^38^. The number following ‘T’ in the nomenclature of the PDX models after ‘TU-BcX- ‘denotes the number of times the tumor had been serially transplanted in mice before the tumor was removed for analysis. PDX tissues were procured through the Louisiana Cancer Research Consortium Biospecimen Core and processed following NIH regulations and institutional guidelines of Tulane University with IRB exemption status. All animal procedures were conducted in compliance with State and Federal laws, standards of the U.S. Department of Health and Human Services, and guidelines established by the Tulane University Animal Care and Use Committee.

TU-BcX-4QATb2 organoids were treated with DMSO or NSC260594 (10 μM), Everolimus (20 μM) and 10 μM NSC260594 + 20 μM Everolimus for 72 h. Media was removed and cells were stained with Calcein-AM (2 μM) and Ethidium homodimer (EthD)-III (5 μM) for 45 min using the PromoKine live/dead staining kit (New York, USA). Calcein-AM can be transported to live cells, Ethidium homodimer binds to DNA of lysed (dead) cells. Organoids were imaged with fluorescence microscopy. The 588 nM excitation channel was used to identify red, ‘dead’ cells, and the 420 nM excitation channel was used to visualize green, ‘live’ cells.

### Statistical analysis

Data are shown as means ± SEM if not otherwise indicated. Two-tailed unpaired Student’s t-test was applied for statistical analysis to compare the two groups of interest and *P *< 0.05 was considered statistically significant unless otherwise stated. Graphical information performed using GraphPad Prism software (GraphPad Software Inc., San Diego, CA).

## Supplementary Information

Supplementary Information.

Supplementary Information.

## Data Availability

All data generated or analyzed during this study are included in this article [and its supplementary information files].
