# Supplementary material for: Targeting Mcl-1 by a small molecule NSC260594 for triple-negative breast cancer therapy
Source: Sci Rep. 2023 Jul 22;13:11843. doi: 10.1038/s41598-023-37058-4 (PMC10363135; doi:10.1038/s41598-023-37058-4)
Supplement: Supplementary file 1 — Supplementary Information. [file 41598_2023_37058_MOESM1_ESM.pdf]

Supplemental Figure. 1

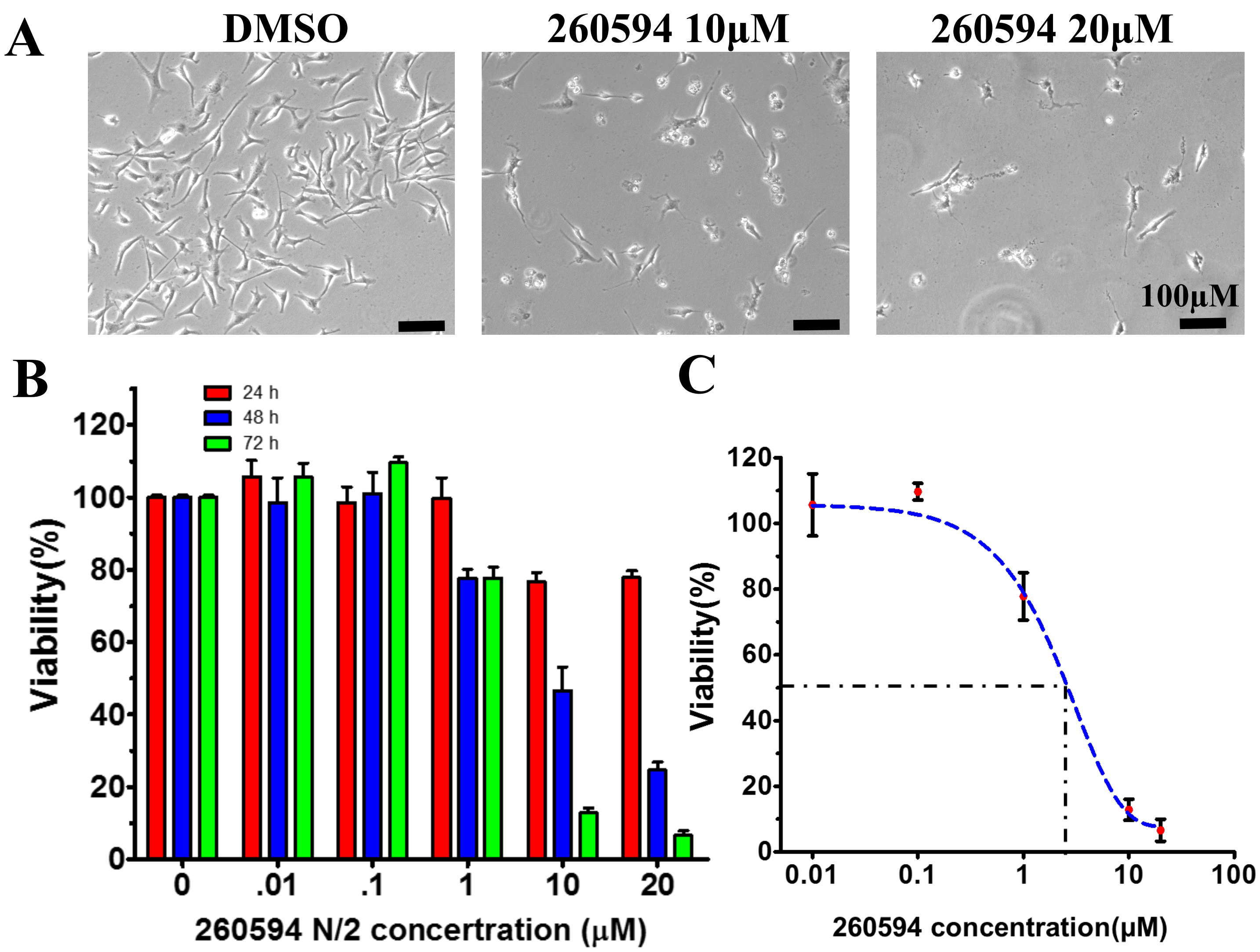

SFig. 1. NSC260594 treatment efficiently killed human TNBC cells. (A) The morphology of MDA-MB231 cells after 260594 treatment for 48h at different doses. (B) 260594 killed MDA-MB231 cells in a dosage and time dependent manner. (C) The IC50 of 260594 appeared to be 4 μM for MDA-MB 231 cells.

Supplemental Figure. 2

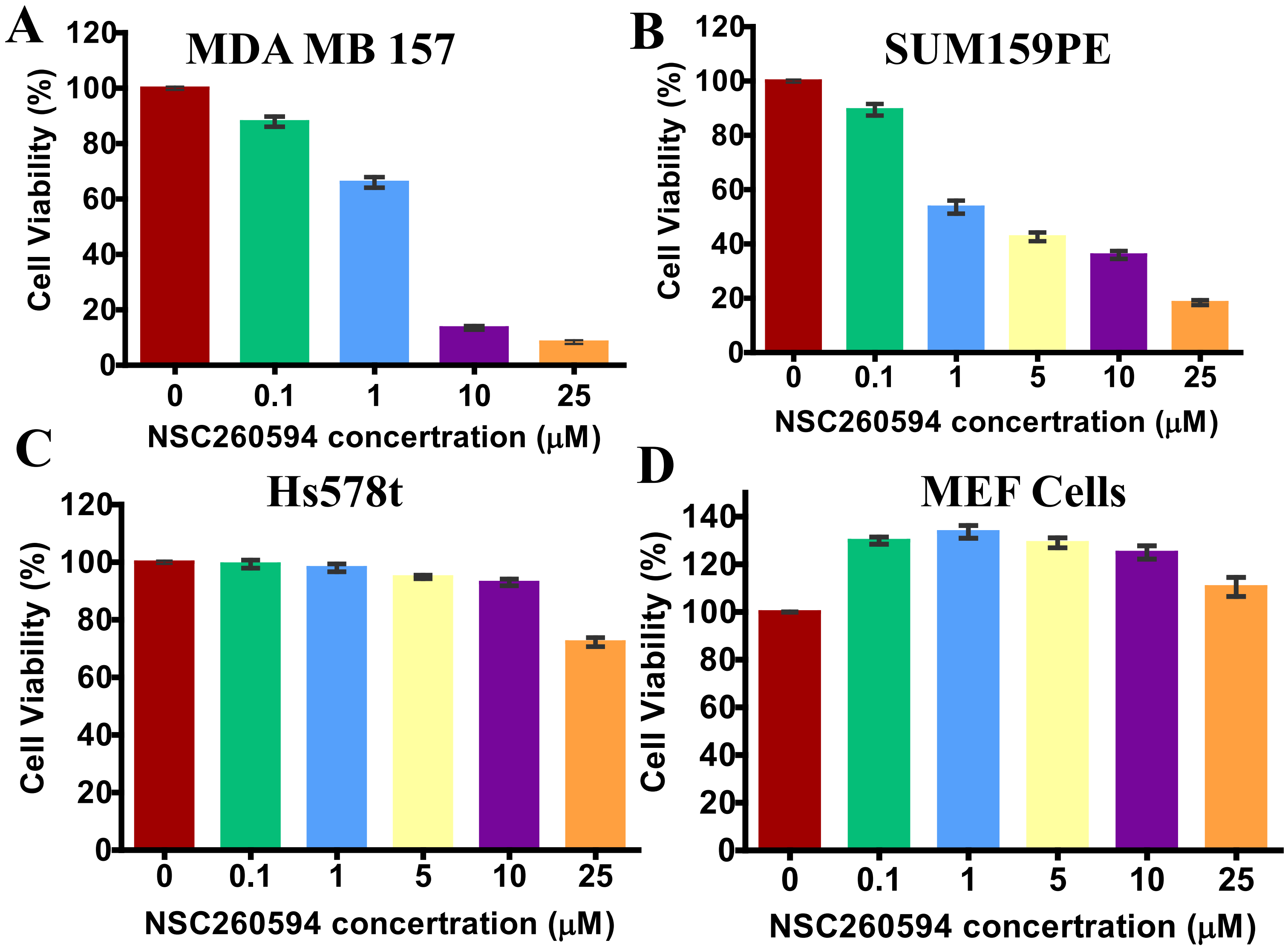

SFig. 2. NSC260594 treatment efficiently killed human TNBC cells. NSC260594 treatment efficiently killed multiple TNBC cell lines in a dose-dependent manner. We treated (A) MDA-MB 157 cells; (B)SUM159PE cells; (C) Hs578t cells; and (D) MEF cells with different dosage of NSC260594 for 72h. Cell viability was determined in quadruplicate using the Cell Counting Kit 8. NSC260594 treatment efficiently killed heterogeneous TNBC cell lines but not mouse embryonic fibroblast (MEF) cells.

Supplemental Figure. 3

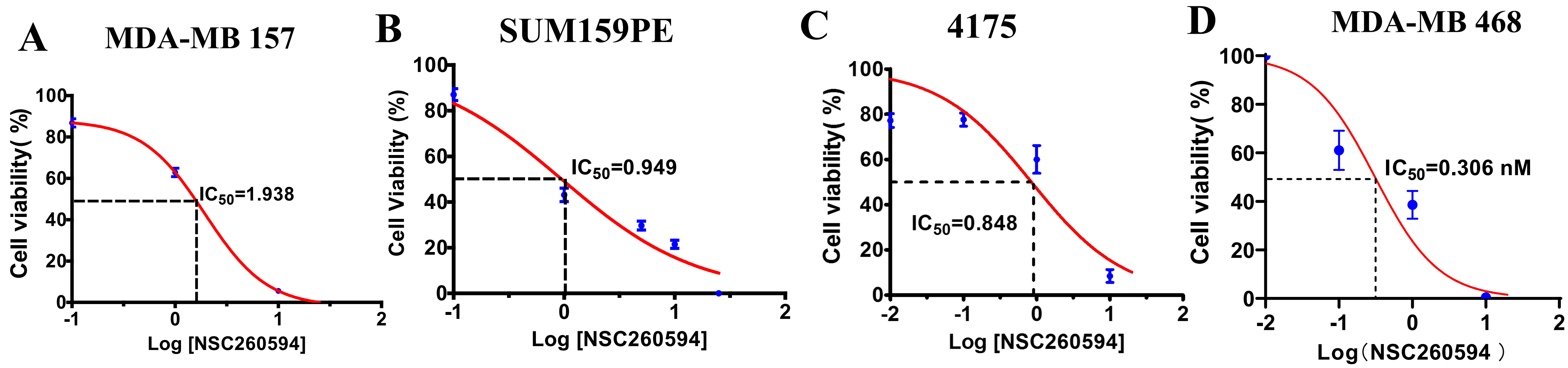

SFig. 3. IC_{50} of NSC 260594 in (A) MDA-MB-157, (B) SUM159PE , (C) 4175 and (D) MDA-MB 468 cells. TNBC cells were seeded (3,000 cells per well) in 96-well plates. Growth medium was replaced with either fresh medium (DMSO as a control) or medium containing the drugs for 72h after overnight growth. Cell viability was determined in quadruplicate using Cell Counting Kit 8. The data analysis performed using Prism software (GraphPad Software).

Supplemental Figure. 4

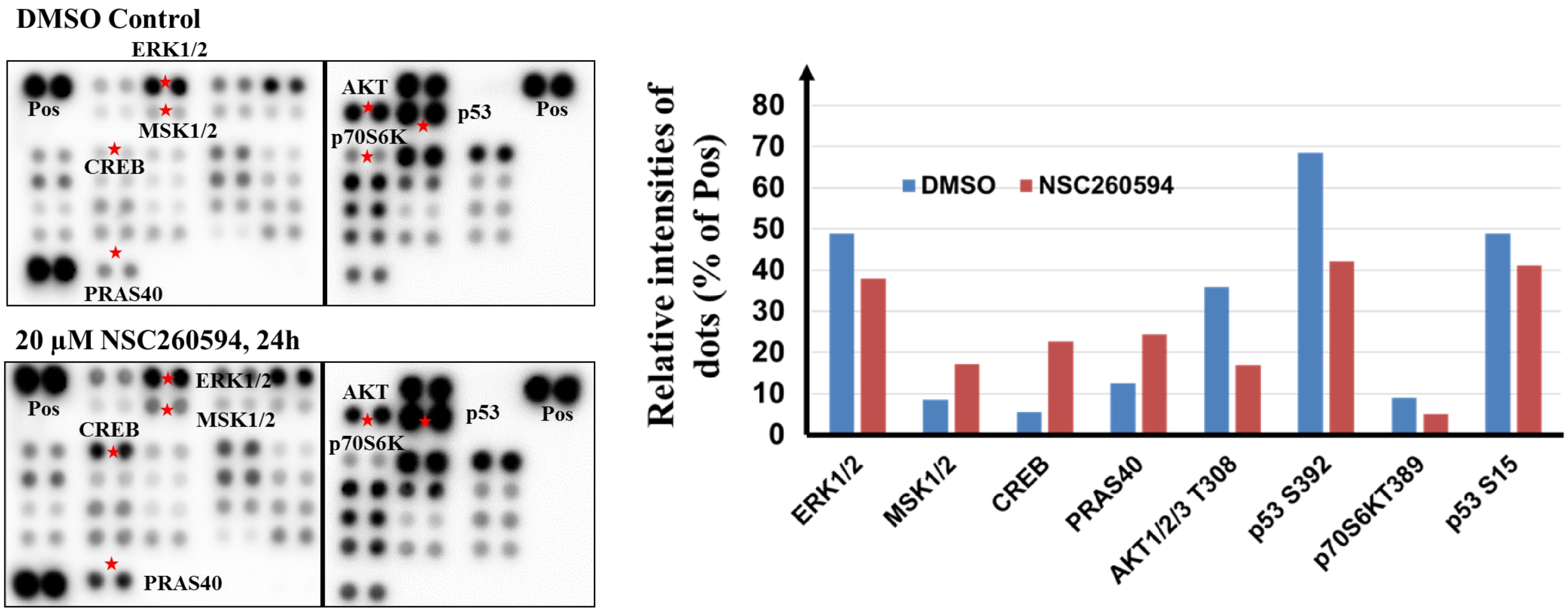

SFig. 4. NSC260594 treatment inhibited ERK and AKT in MDA-MB 231 cells. MDA-MB-231 cells were grown in DMEM containing 10% fetal bovine serum (FBS) and penicillin/streptomycin, cells were with DMSO, or 20µM NSC260594 for 24h. To determine which receptor tyrosine kinases (RTKs) are targeted by NSC260594, the R&D Human RTK Phosphorylation Antibody Array Kit was used. The relative intensities of the duplicated spots were normalized to positive control spots. Values represent the mean of duplicate spots for each protein after normalization.

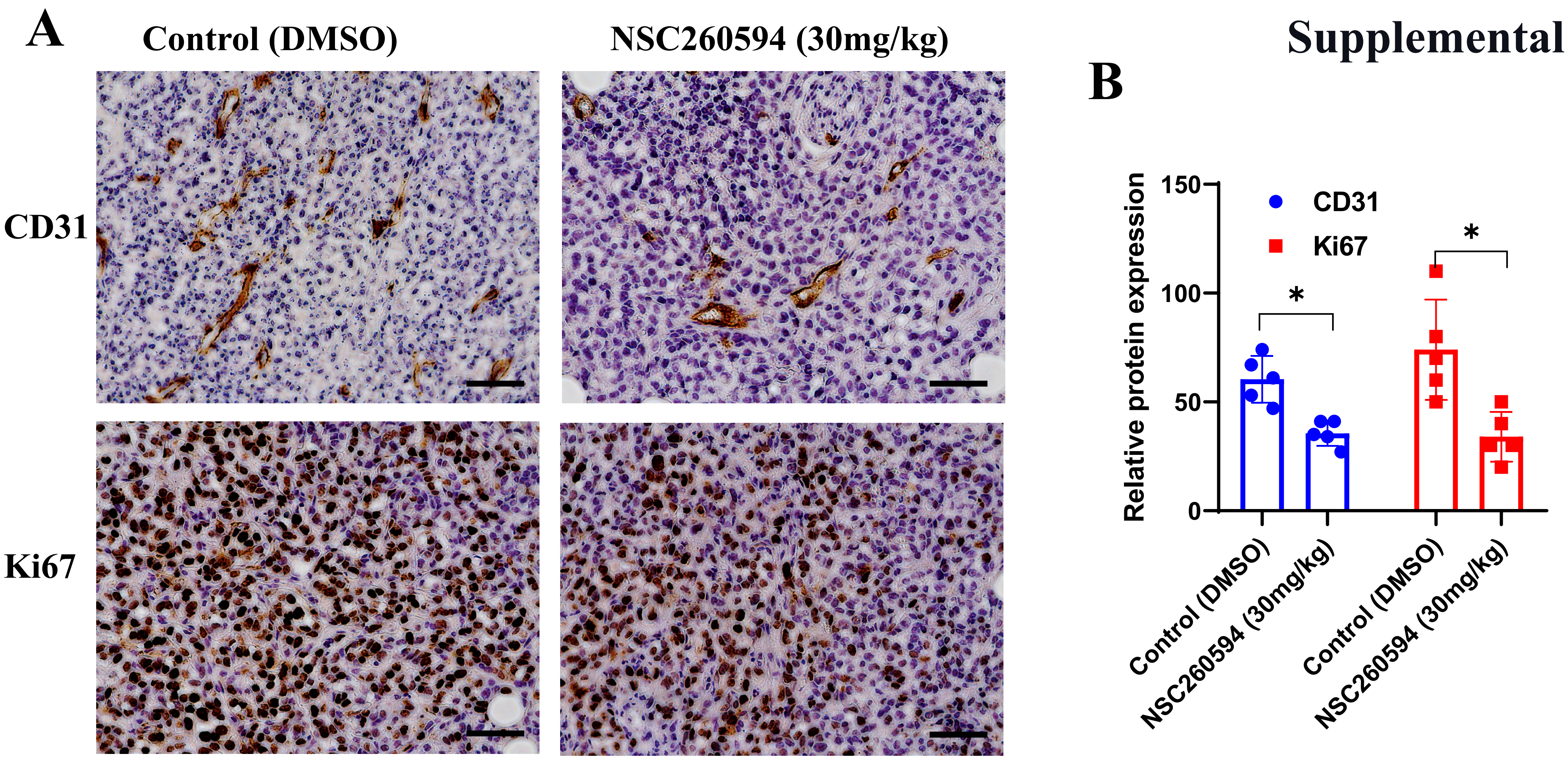

SFig. 5 NSC 260594 treatment reduced the CD31 and Ki-67 expression in vivo. (A) Immunohistochemical staining of CD31, and Ki-67 of the mice injected with MDA-MB 231 cells. **(B)** Quantification of the IHC staining (n = 5). Data are expressed as the number of CD31, and Ki67 positive cells per field. Data were expressed as the mean ± SD per field. Statistically significant values of *p < 0.05 were determined compared with the control. Scale bar, 100 µm.

Supplemental Figure 6

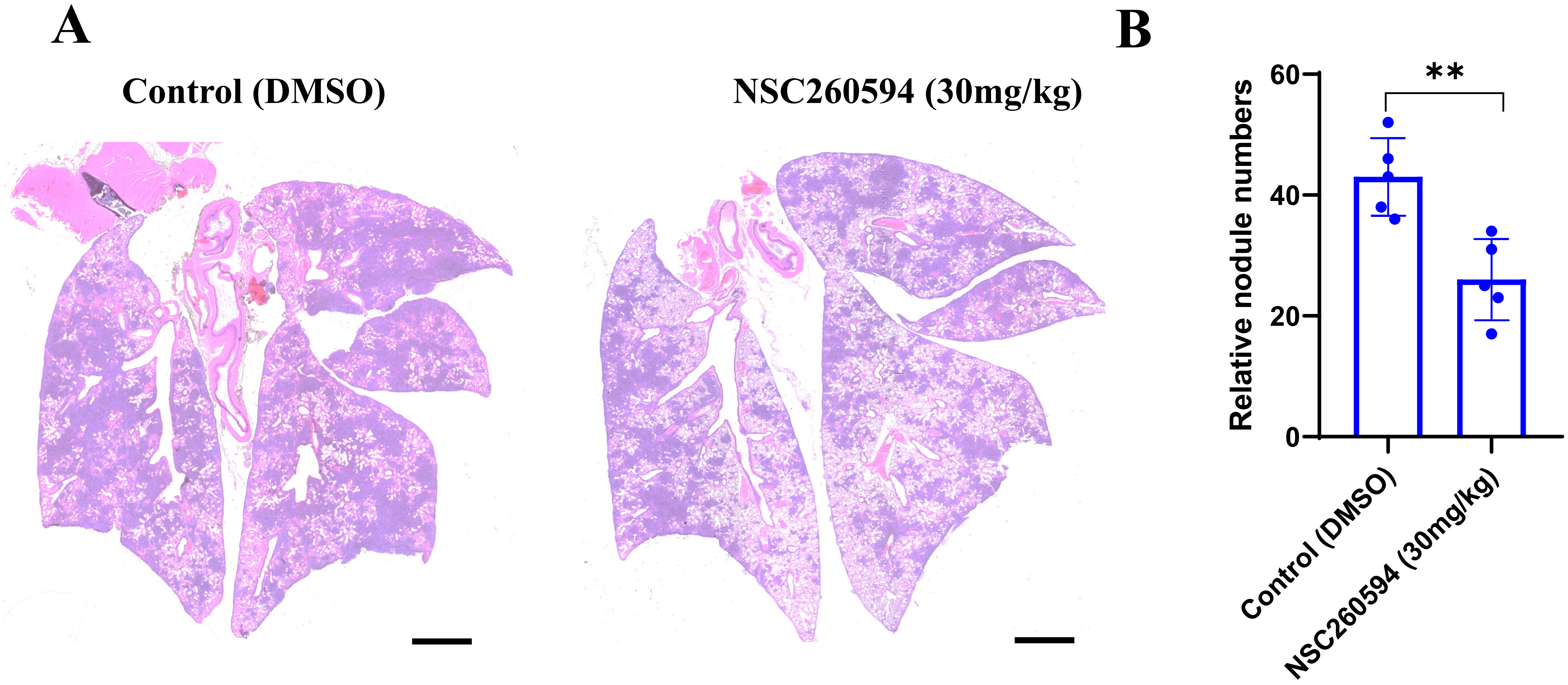

SFig. 6 NSC 260594 treatment suppressed lung metastasis. (A) Histological analysis of tumor metastasis of MDA-MB 231 cells in the lung of NSG mice (Control (DMSO), (n = 5) and NSC 260594 (30 mg/kg body weight), (n = 5)). **(B)** Mean metastatic nodule count in each group at the experimental endpoint was compared. Lung metastasis (sections of lungs were stained with H&E). Scale bar, 100 μm.
