# Supplementary material for: Targeting Mcl-1 by a small molecule NSC260594 for triple-negative breast cancer therapy
Source: Sci Rep. 2023 Jul 22;13:11843. doi: 10.1038/s41598-023-37058-4 (PMC10363135; doi:10.1038/s41598-023-37058-4)
Supplement: Supplementary file 2 — Supplementary Information. [file 41598_2023_37058_MOESM2_ESM.pdf]

Fig. 2

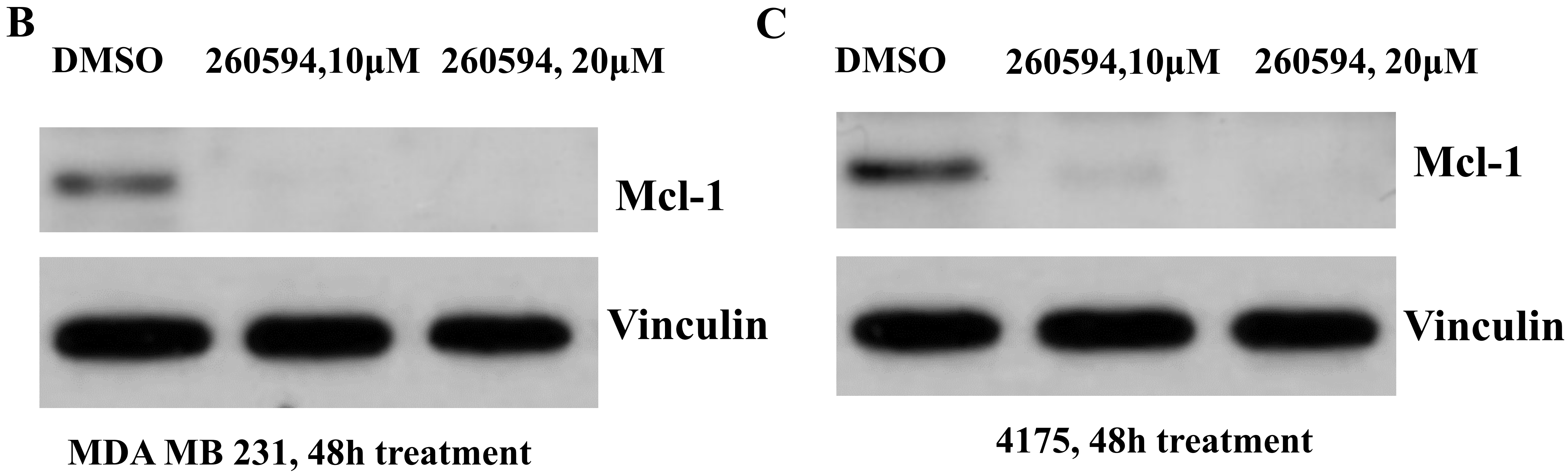
B
DMSO
260594,10μM
260594, 20μM
Mcl-1
Vinculin
MDA MB 231, 48h treatment
C
DMSO
260594,10μM
260594, 20μM
Mcl-1
Vinculin
4175, 48h treatment

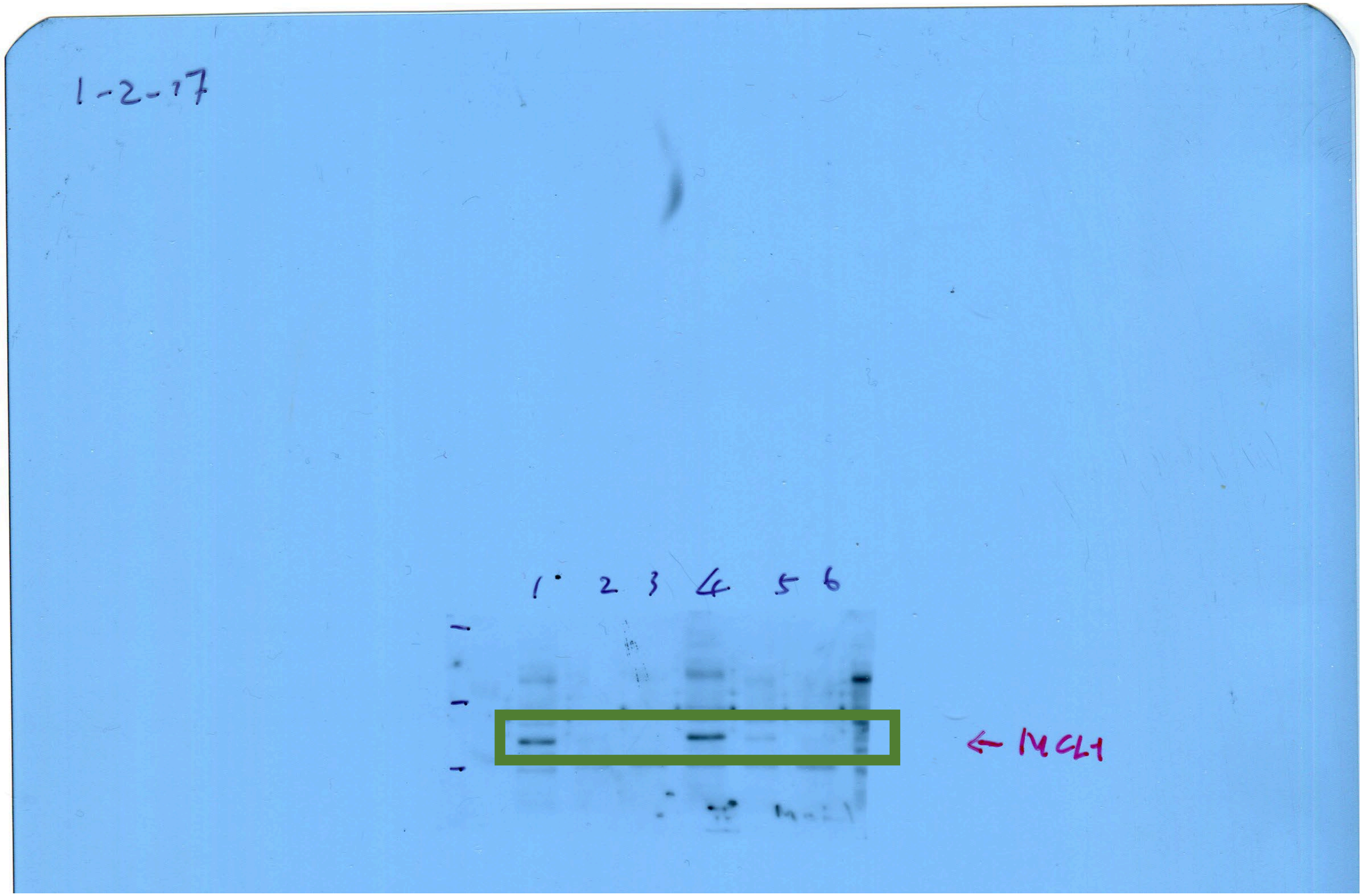

Lanes 1, 2, 3; Figure 2B- Mcl1; **DMSO 260594,10µM 260594, 20µM---- MDA MB 231**

Lanes 4, 5, 6- Figure 2C –Mcl1 **DMSO 260594,10µM 260594, 20µM--- 4175 cells**

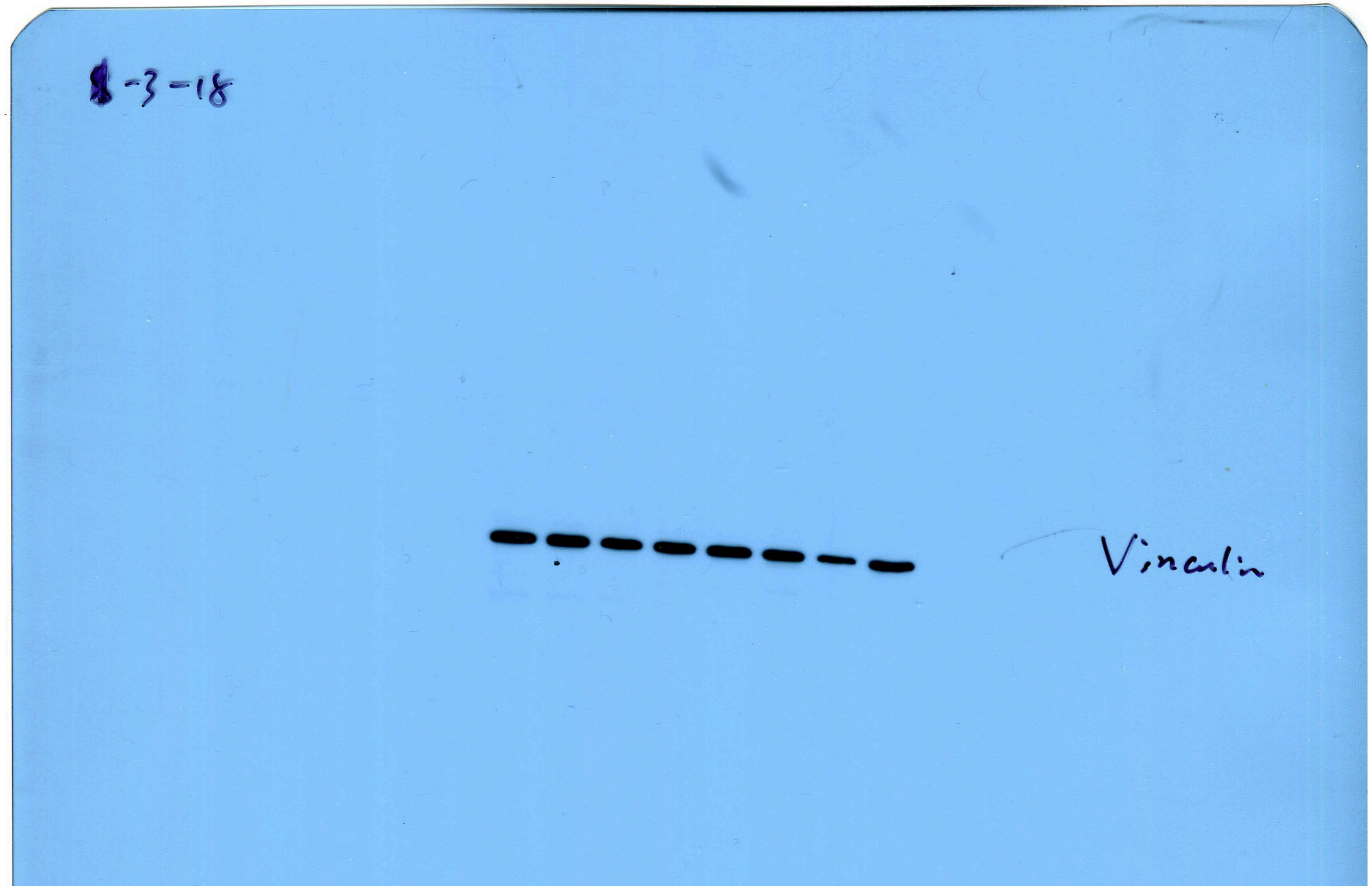

Figure 2B. Vinculin: First three lanes from left to right: MDA MB 231;
Figure 2C. Vinculin: Next three lanes (4 thru 6): 4175 cells

Fig. 3

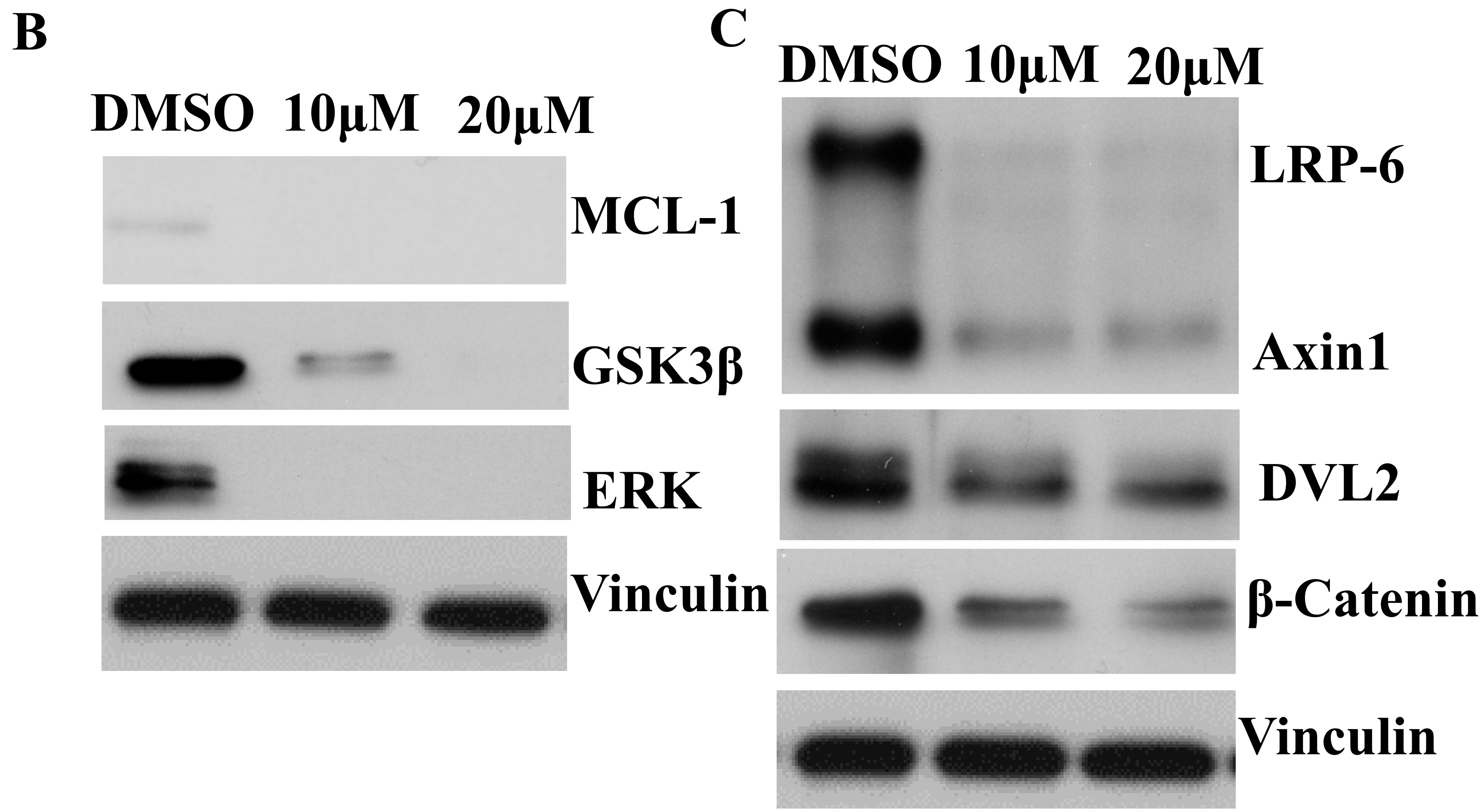

MDA MB-231, 260594 48h treatment

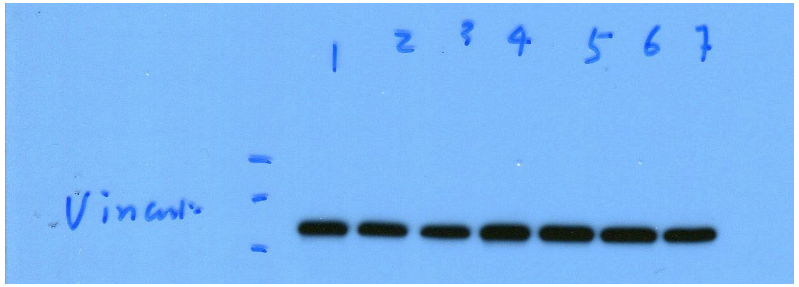

Figure 3B. Vinculin: First three lanes from left to right (1 thru 3)
Figure 3C. Vinculin: Next three lanes (4 thru 6)

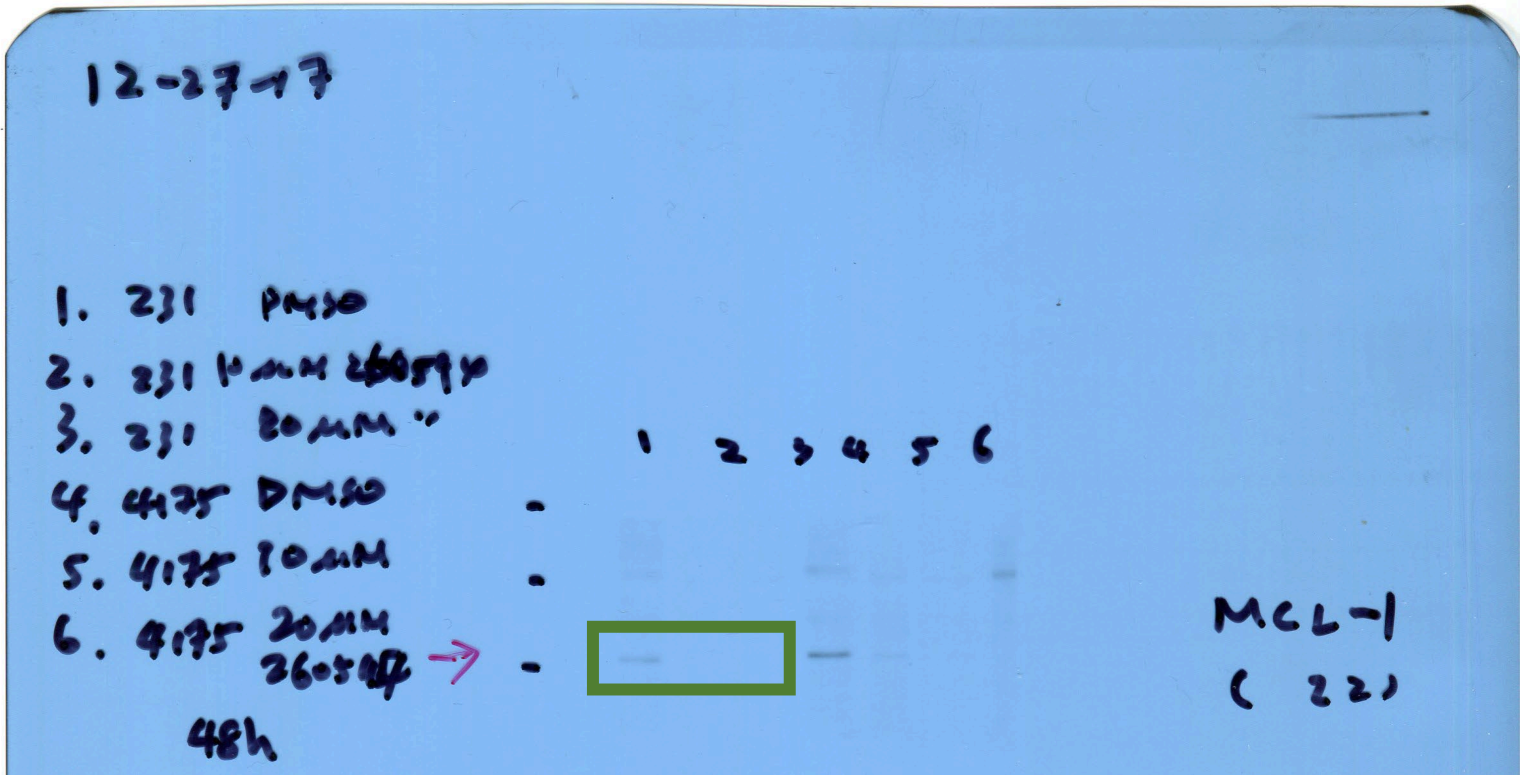

Figure 3B- Mcl 1

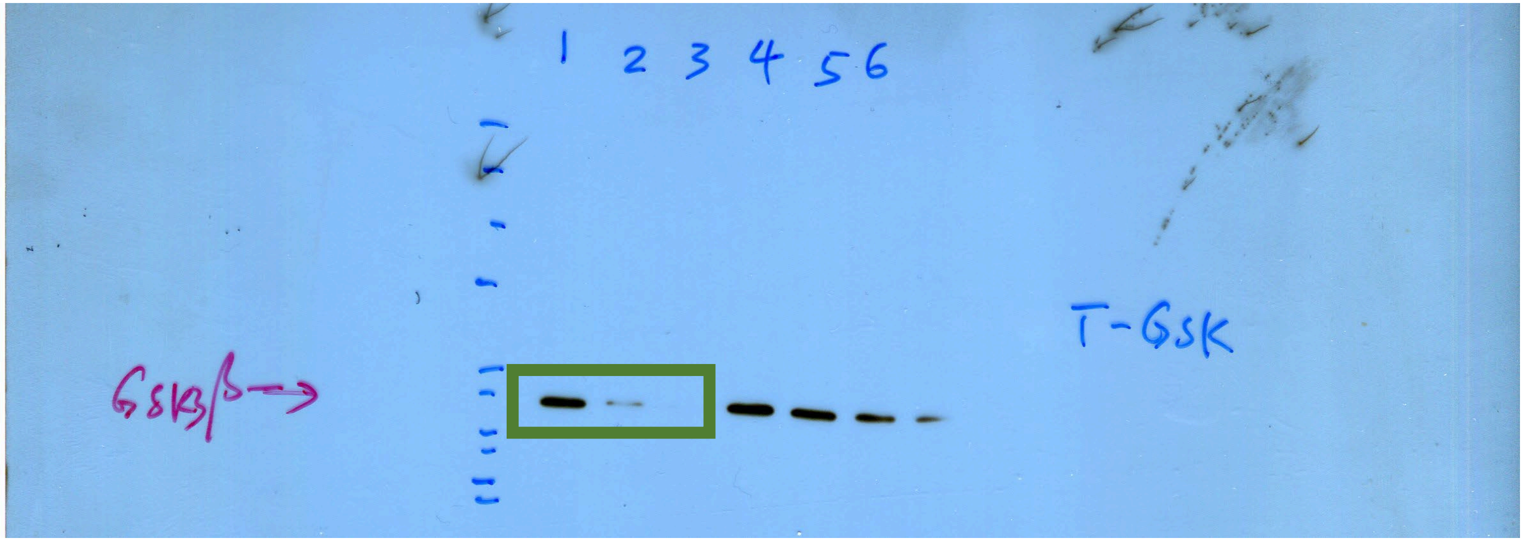

Figure 3B- GSK3beta

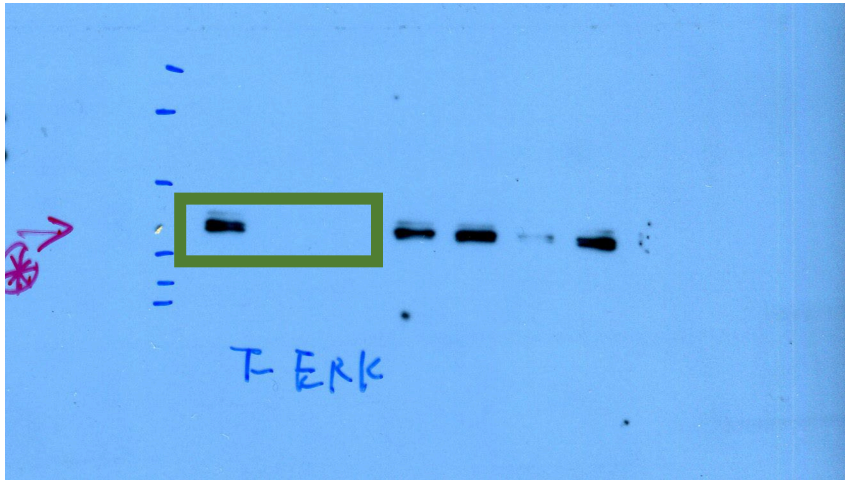

Figure 3B- Erk

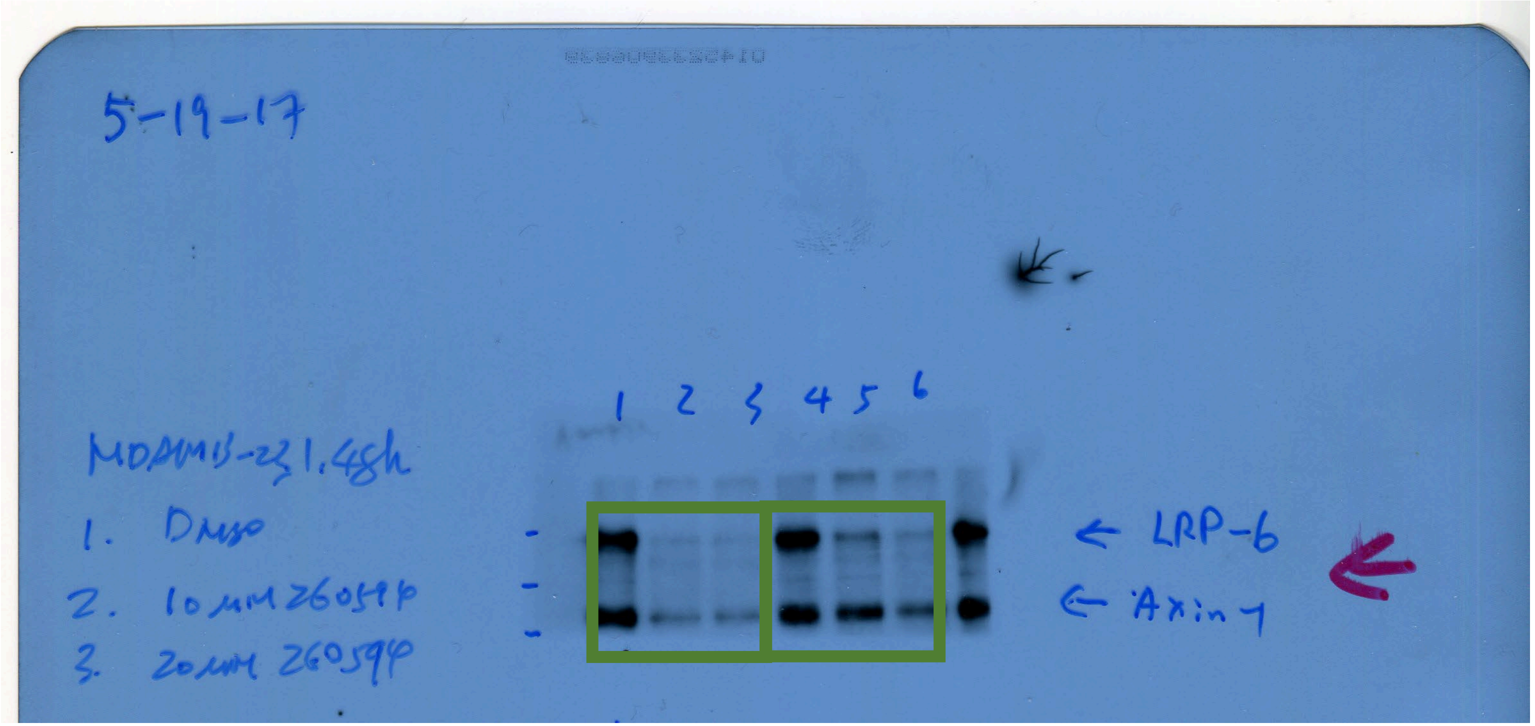

Figure 3C- LRP6 and Axin1

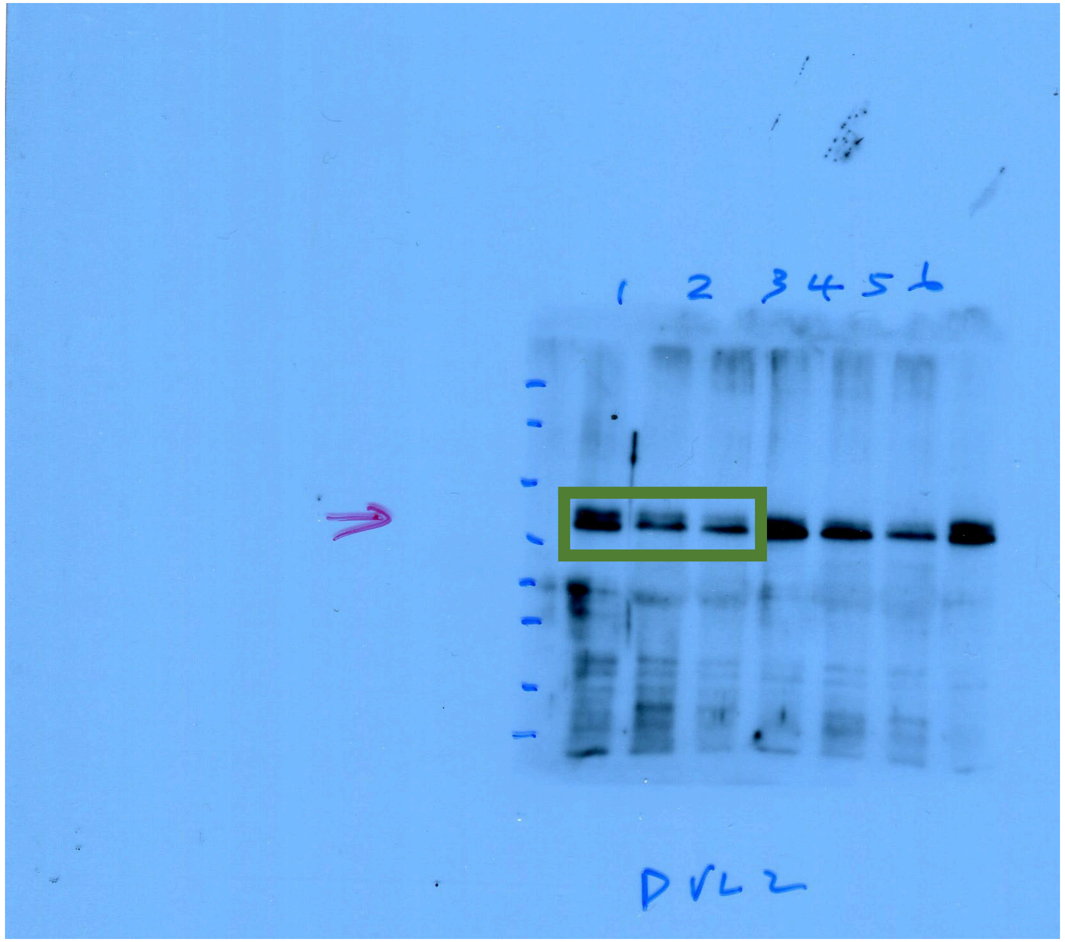

Figure 3C- DVL2

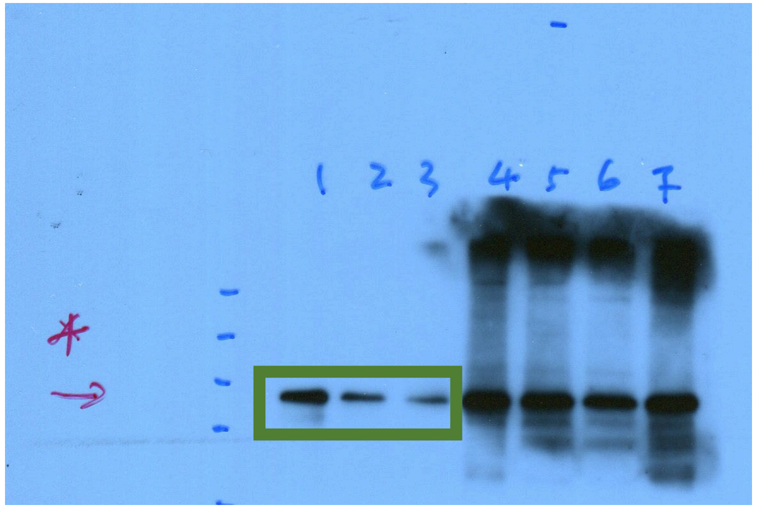

Figure 3C: β Catenin

Fig. 4

C

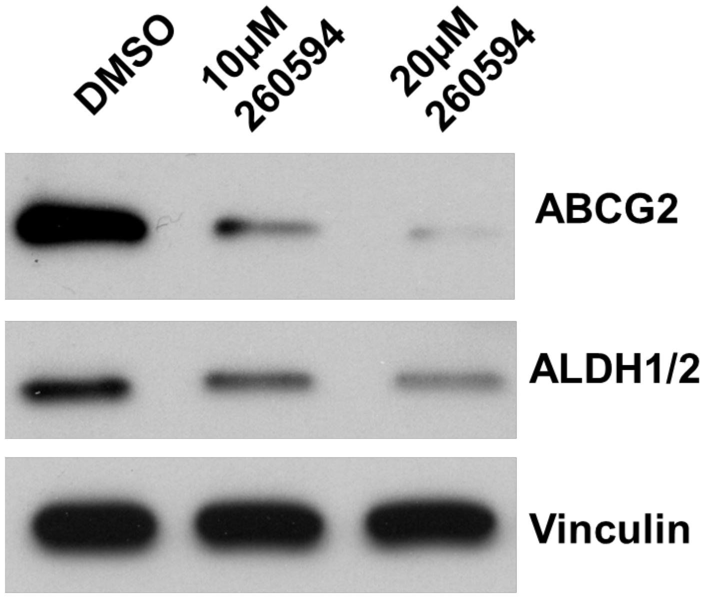

MDA-MB 231, 48h treatment

D

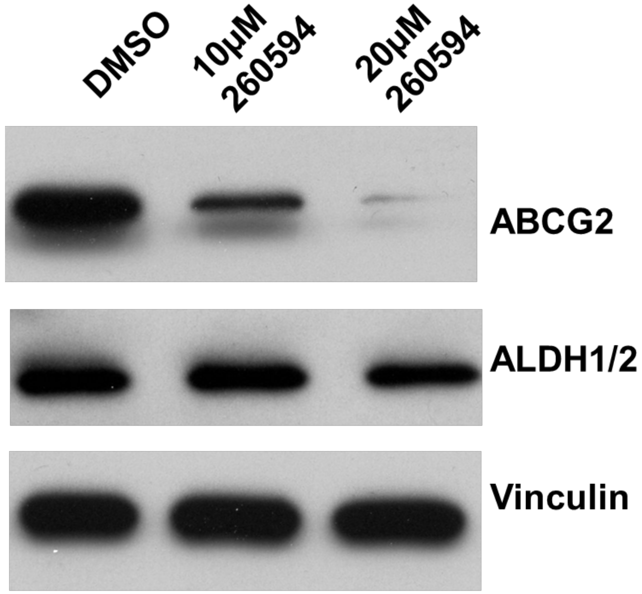

4175, 48h treatment

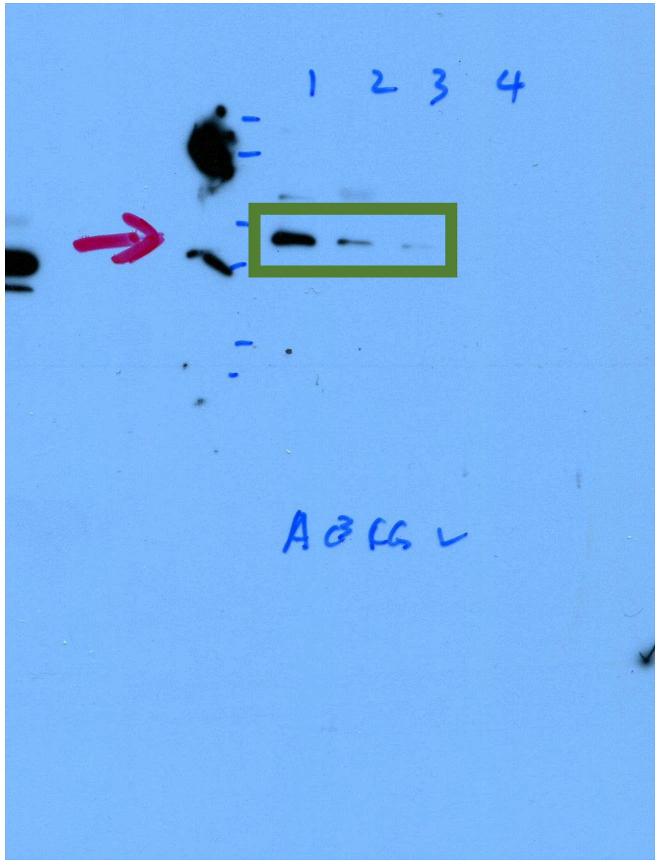

Figure 4C ABCG2

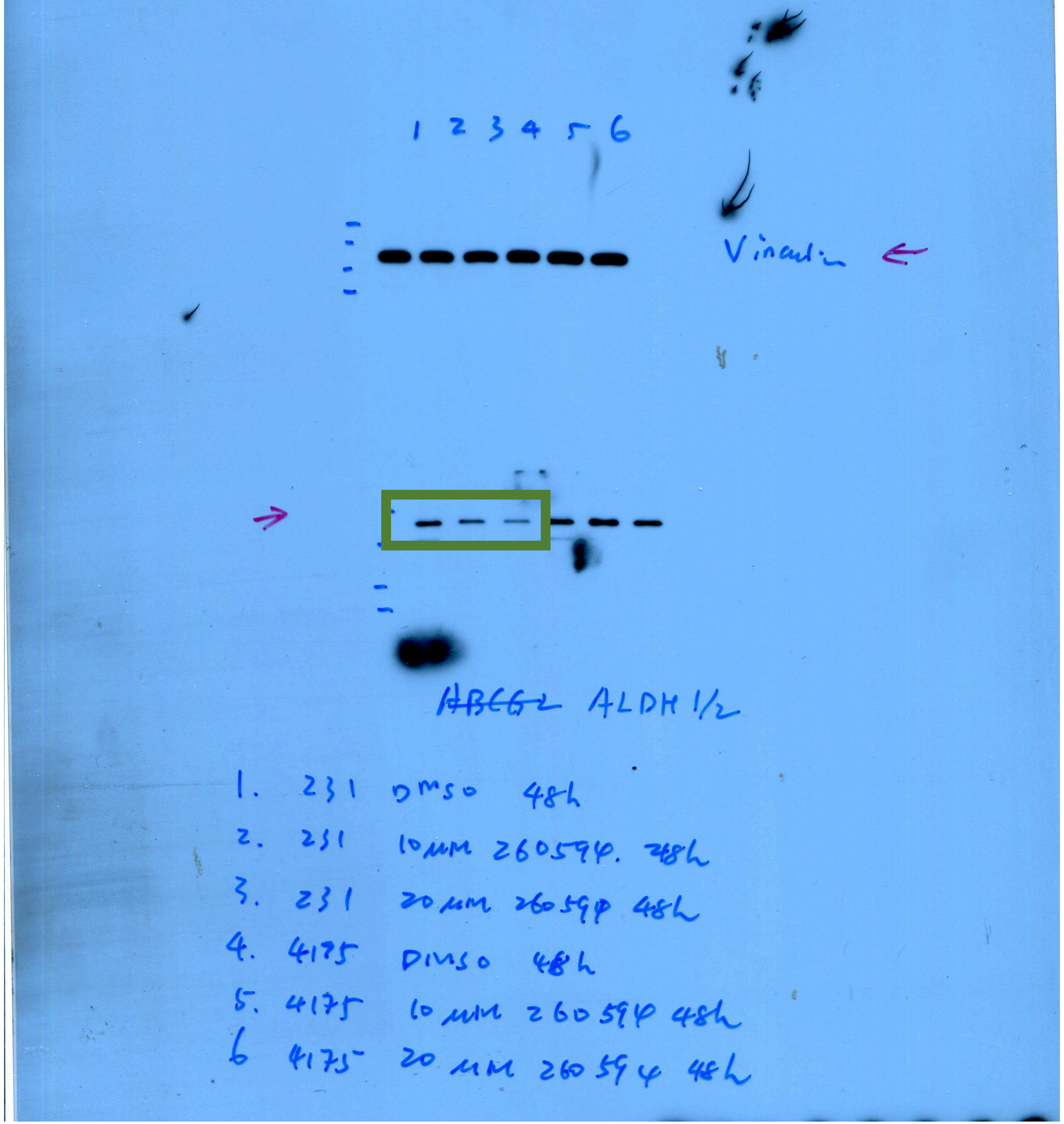

Figure 4C- ALDH ½

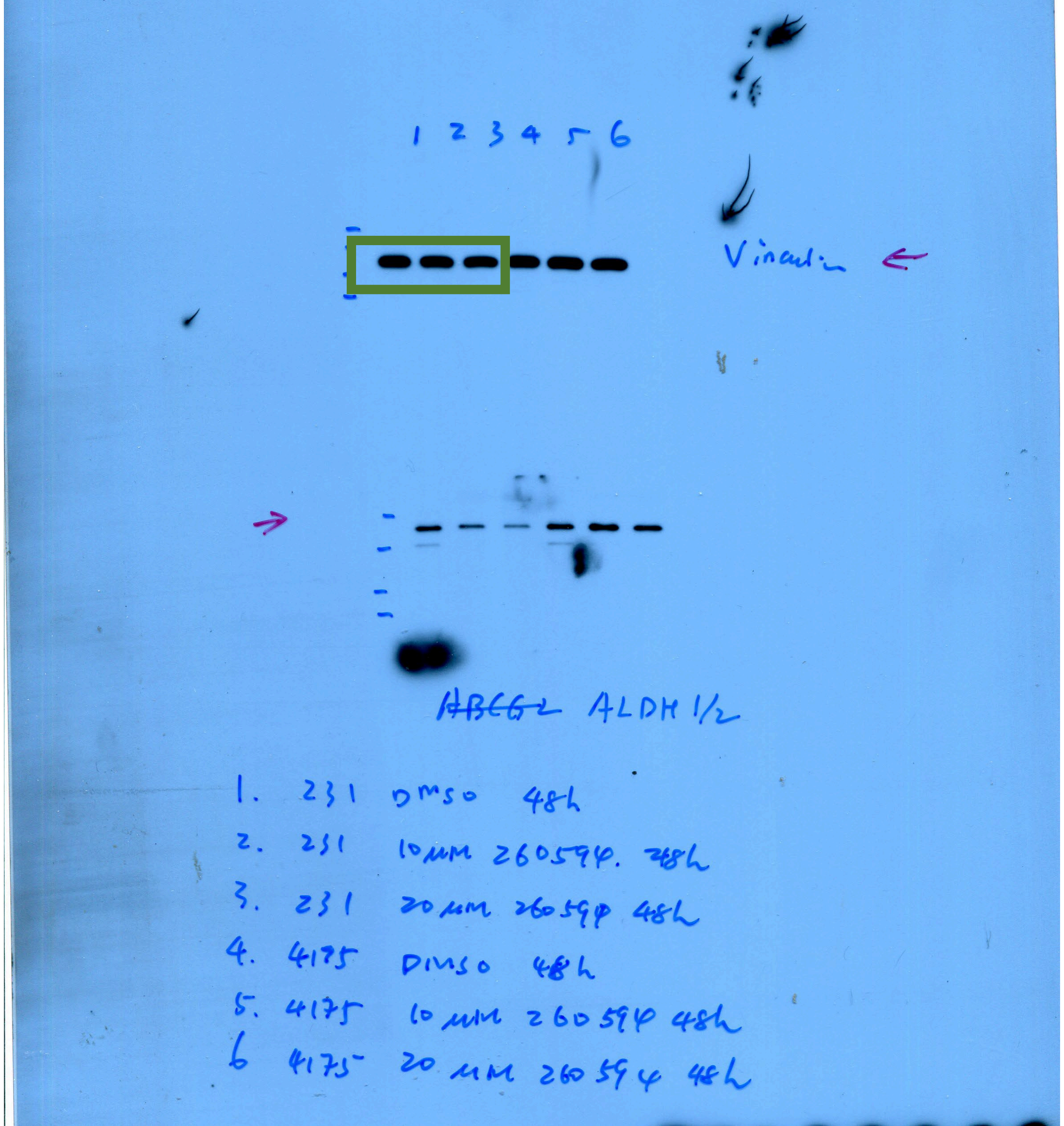

Figure 4C- Vinculin

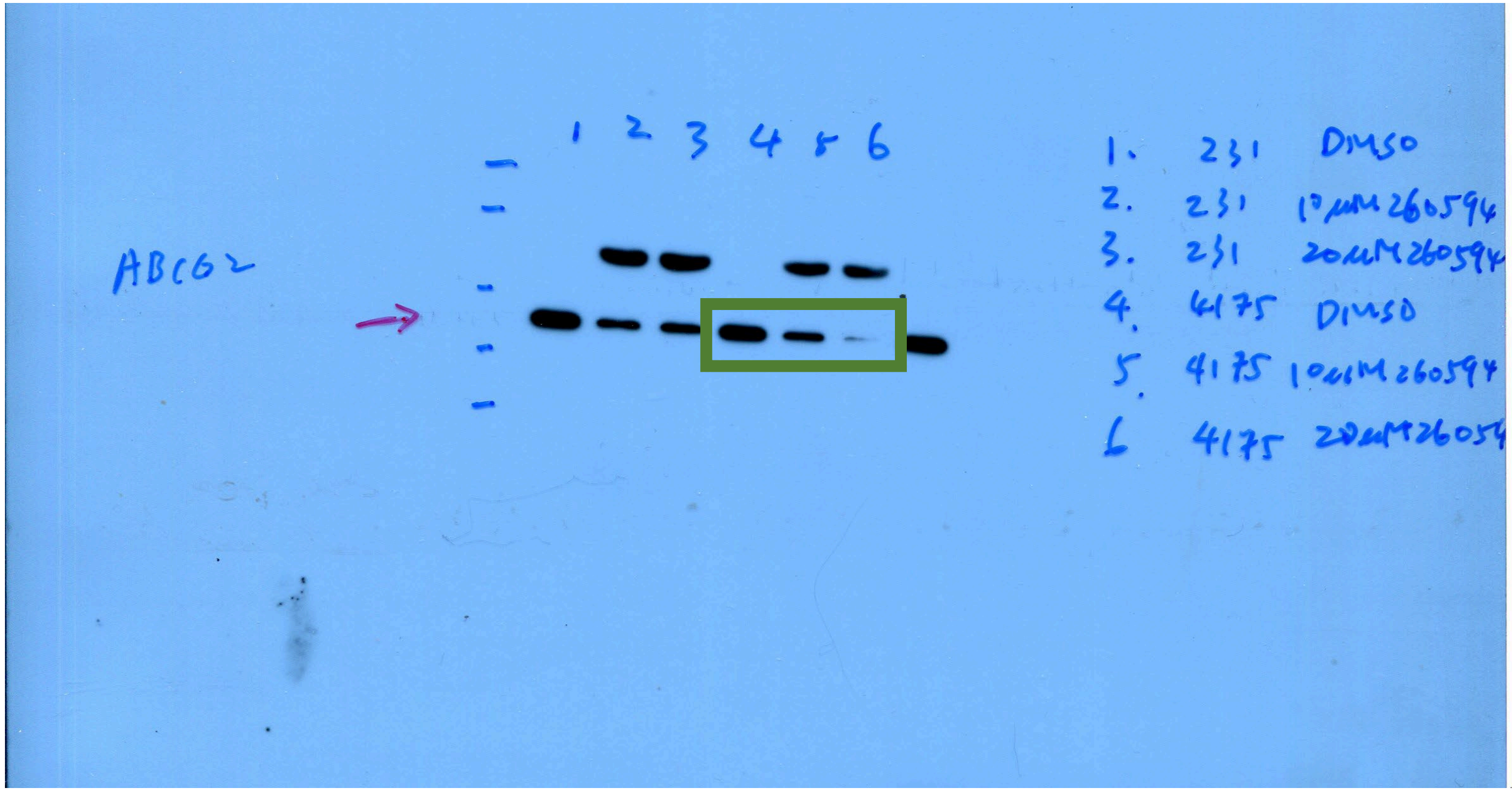

Figure 4D- ABCG2

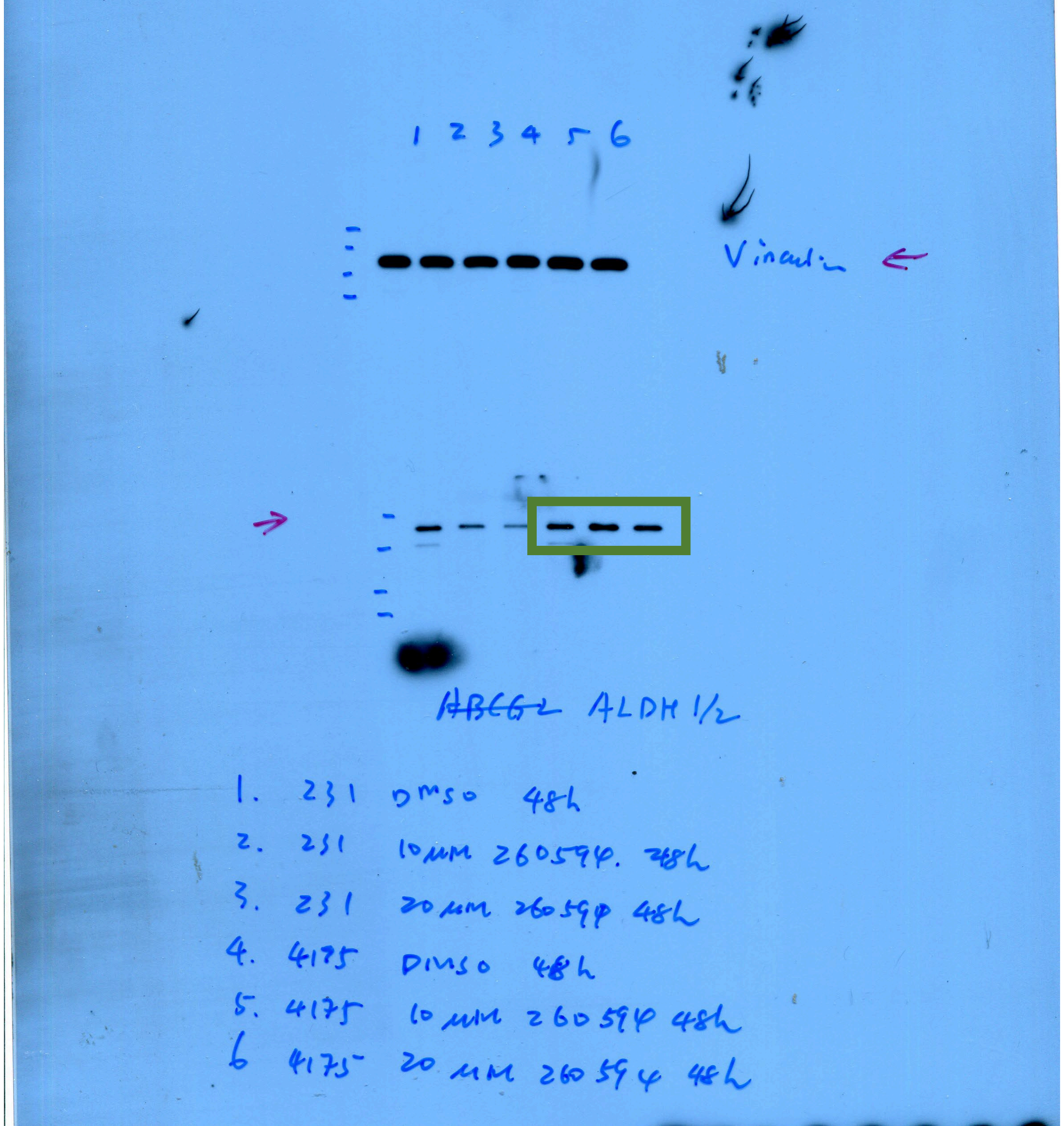

Figure 4D ALDH1/2

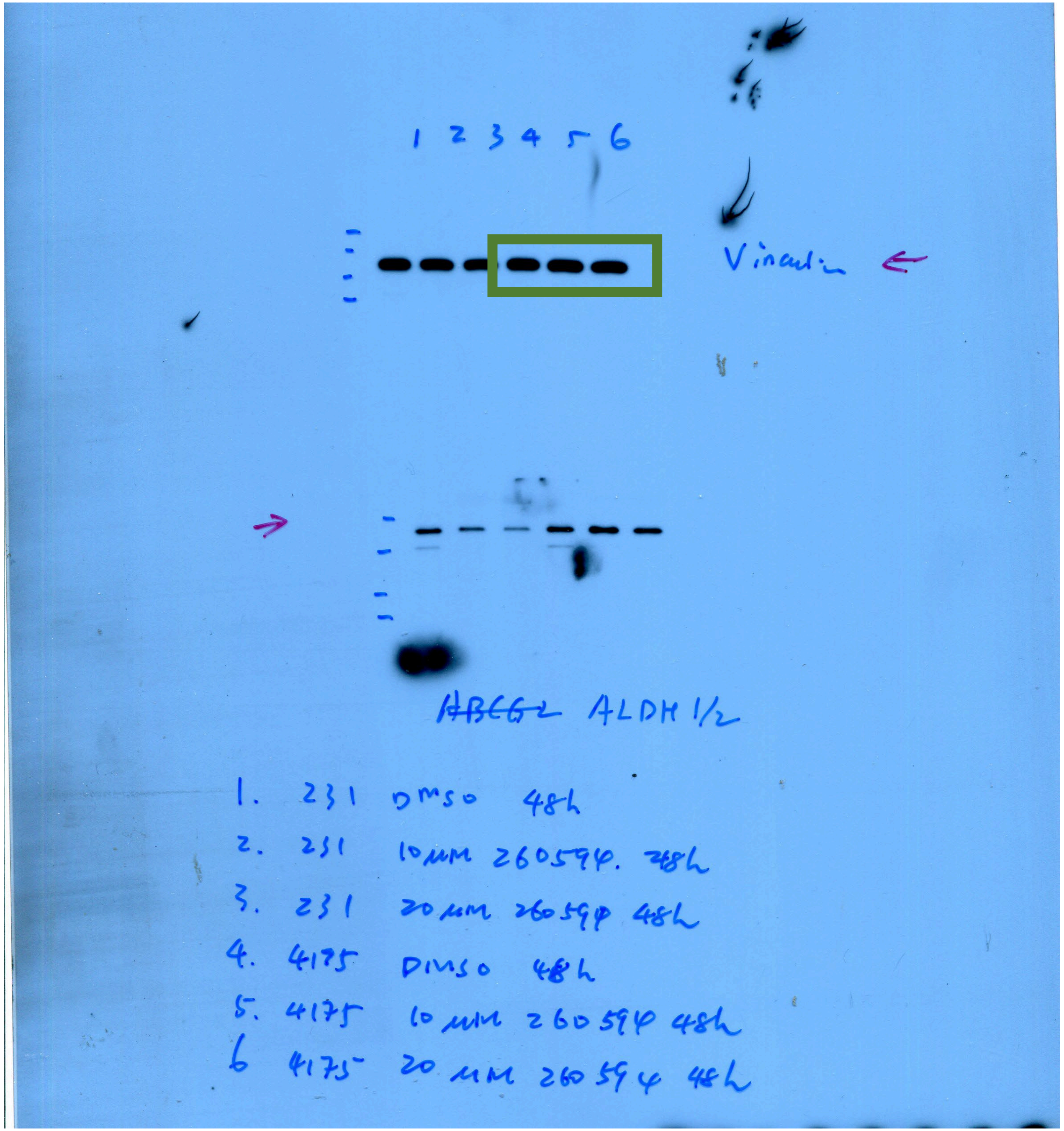

Figure 4D vinculin

Fig. 5

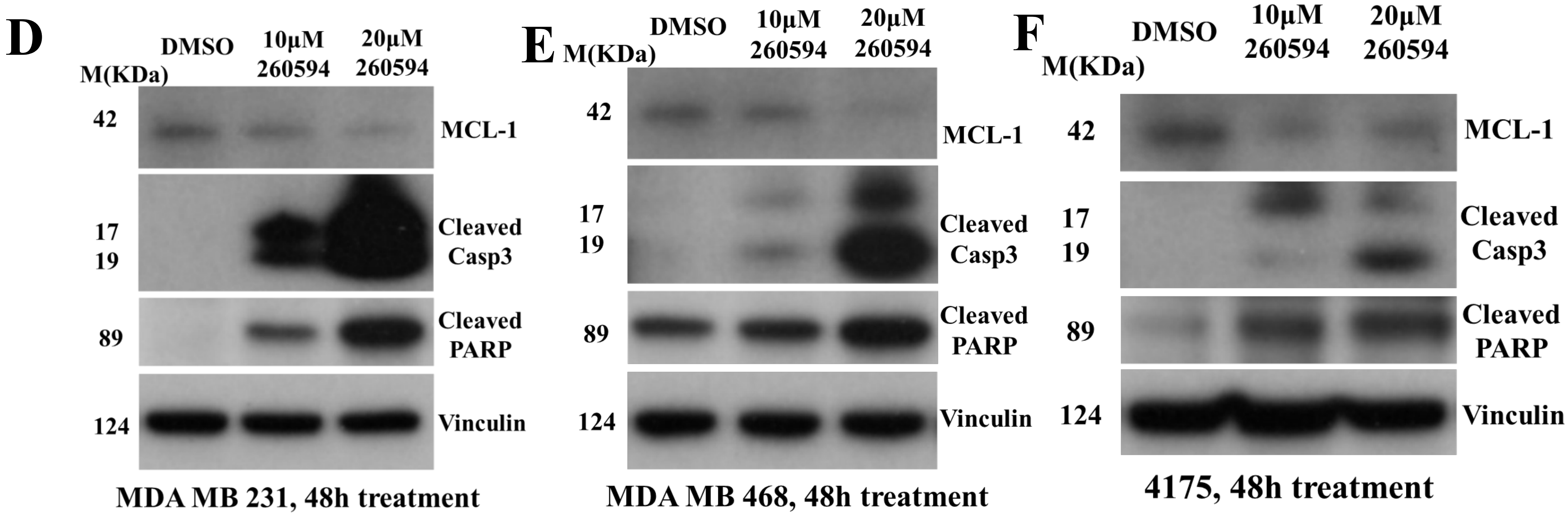

D
M(KDa)
DMSO
10μM 260594
20μM 260594
42
MCL-1
17
19
Cleaved Casp3
89
Cleaved PARP
124
Vinculin
MDA MB 231, 48h treatment
E
M(KDa)
DMSO
10μM 260594
20μM 260594
42
MCL-1
17
19
Cleaved Casp3
89
Cleaved PARP
124
Vinculin
MDA MB 468, 48h treatment
F
M(KDa)
DMSO
10μM 260594
20μM 260594
42
MCL-1
17
19
Cleaved Casp3
89
Cleaved PARP
124
Vinculin
4175, 48h treatment

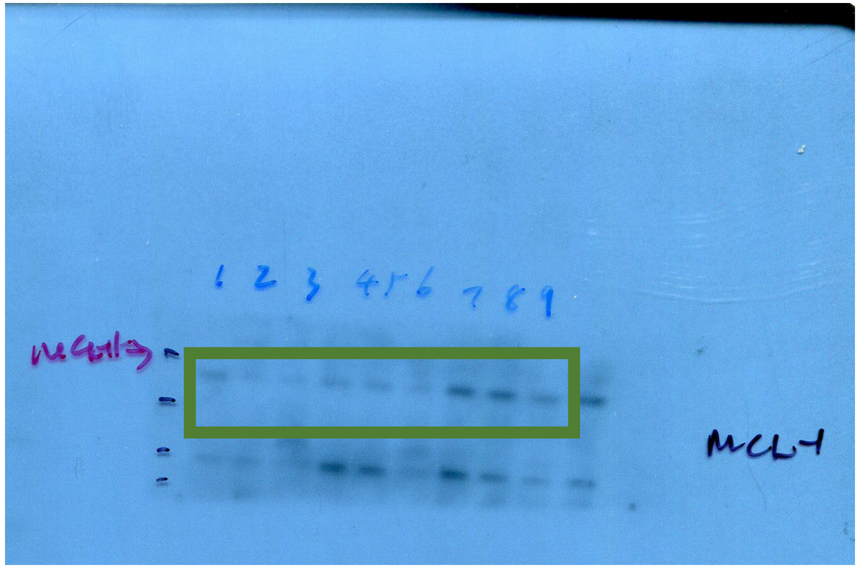

Figure 5D Mcl1- lanes 1,2, 3
Figure 5E Mcl1- lanes 4,5,6
Figure 5F Mcl1- lanes 7,8,9

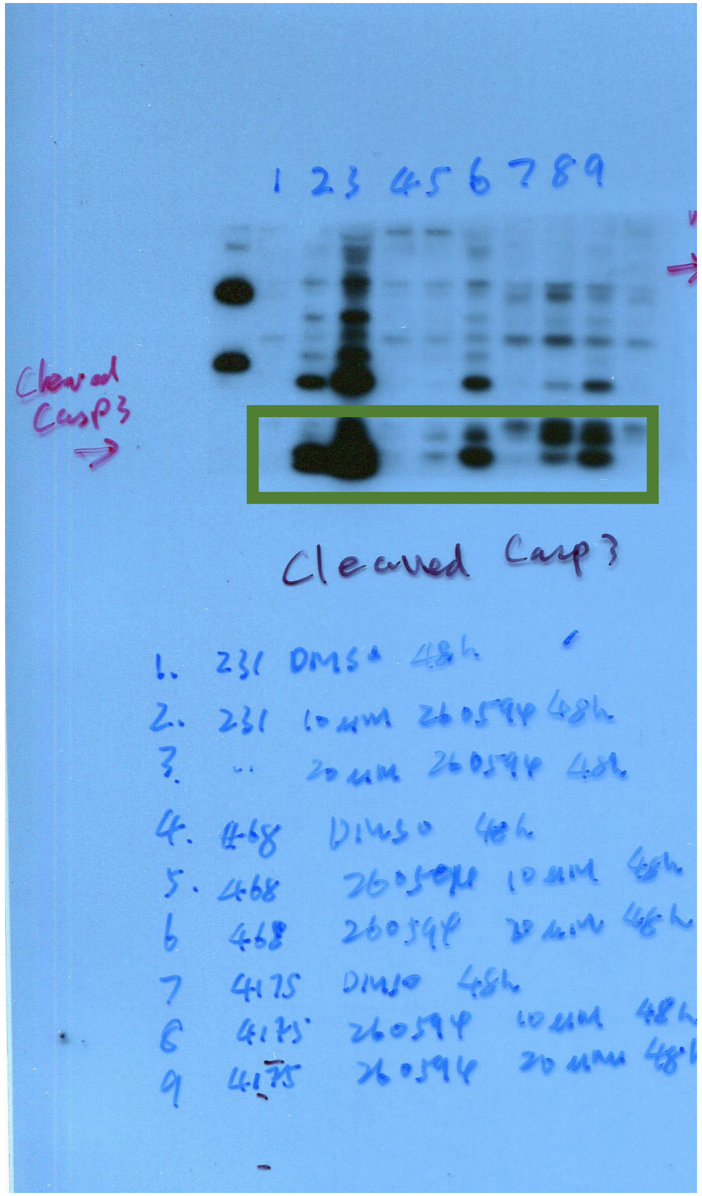

Figure 5D cleaved casp3- lanes 1,2, 3
Figure 5E cleaved casp3- lanes 4,5,6
Figure 5F cleaved casp3- lanes 7,8,9

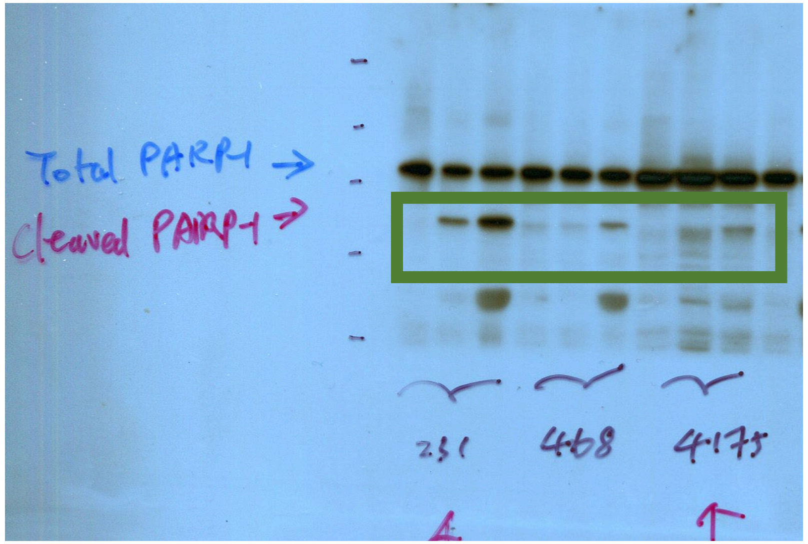

Figure 5D cleaved Parp- lanes 1,2, 3 MDA MB 231
Figure 5E cleaved Parp- lanes 4,5,6 MDA MB 468
Figure 5F cleaved Parp- lanes 7,8,9 4175

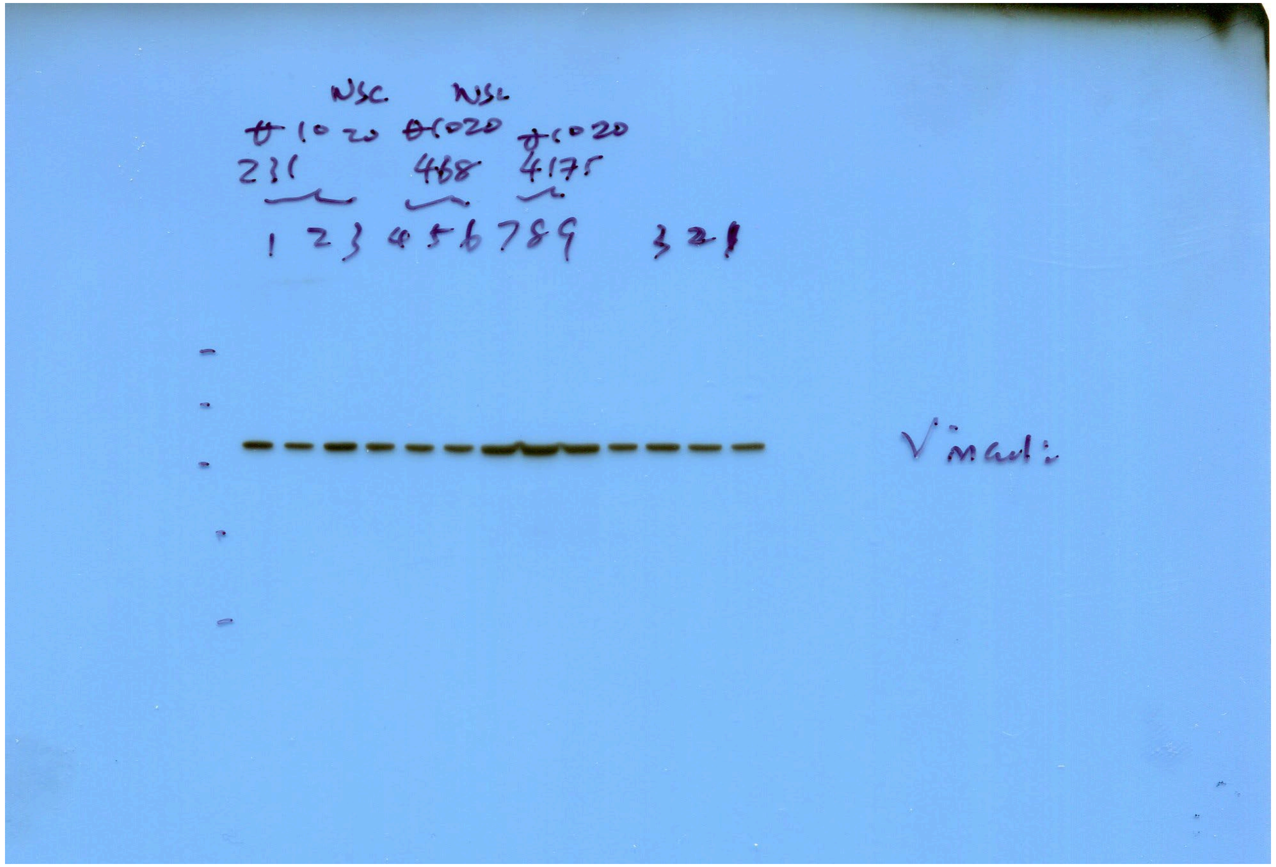

Figure 5D Vinculin lanes 1,2, 3
Figure 5E Vinculin lanes 4,5,6
Figure 5F Vinculin lanes 7,8,9

Figure 6D

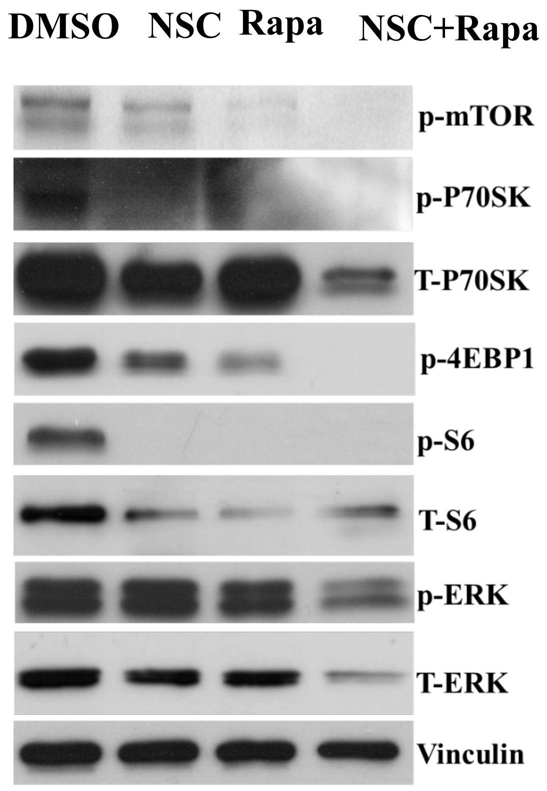
DMSO
NSC
Rapa
NSC+Rapa
p-mTOR
p-P70SK
T-P70SK
p-4EBP1
p-S6
T-S6
p-ERK
T-ERK
Vinculin

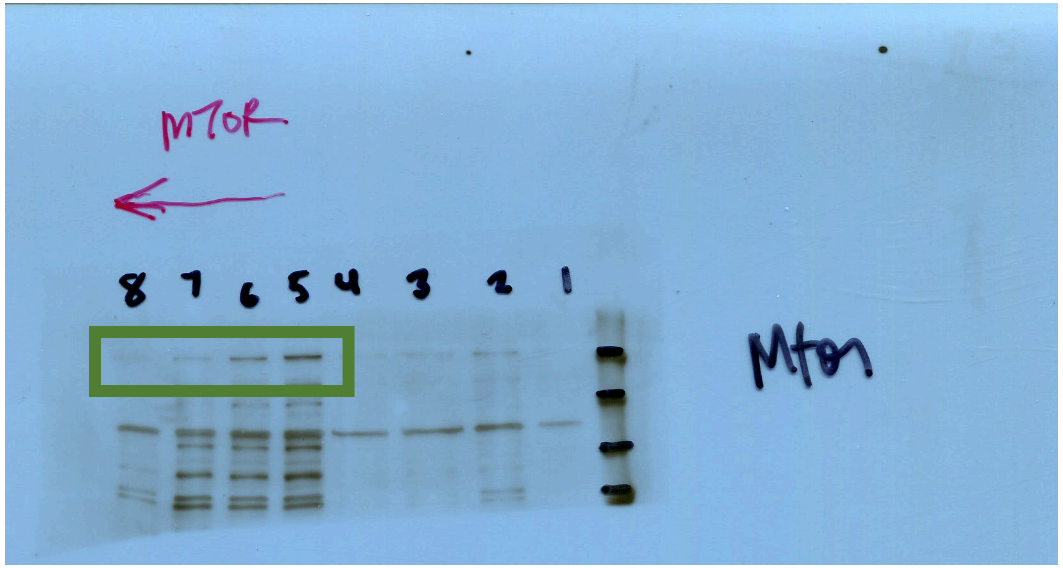

Figure 6D mTOR lanes 5,6,7,8

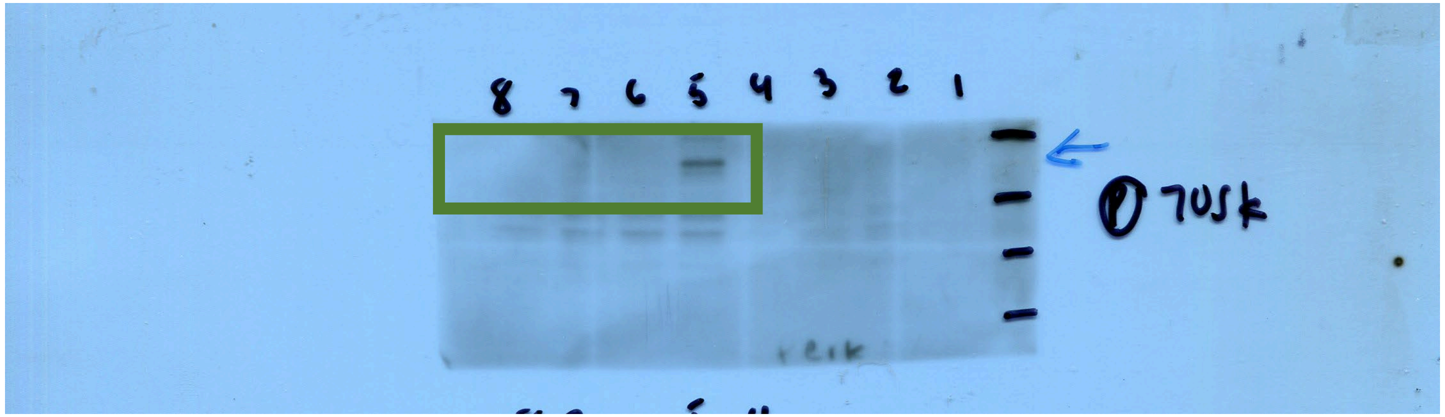

Figure 6D- p-P70SK- lanes 5, 6, 7, 8

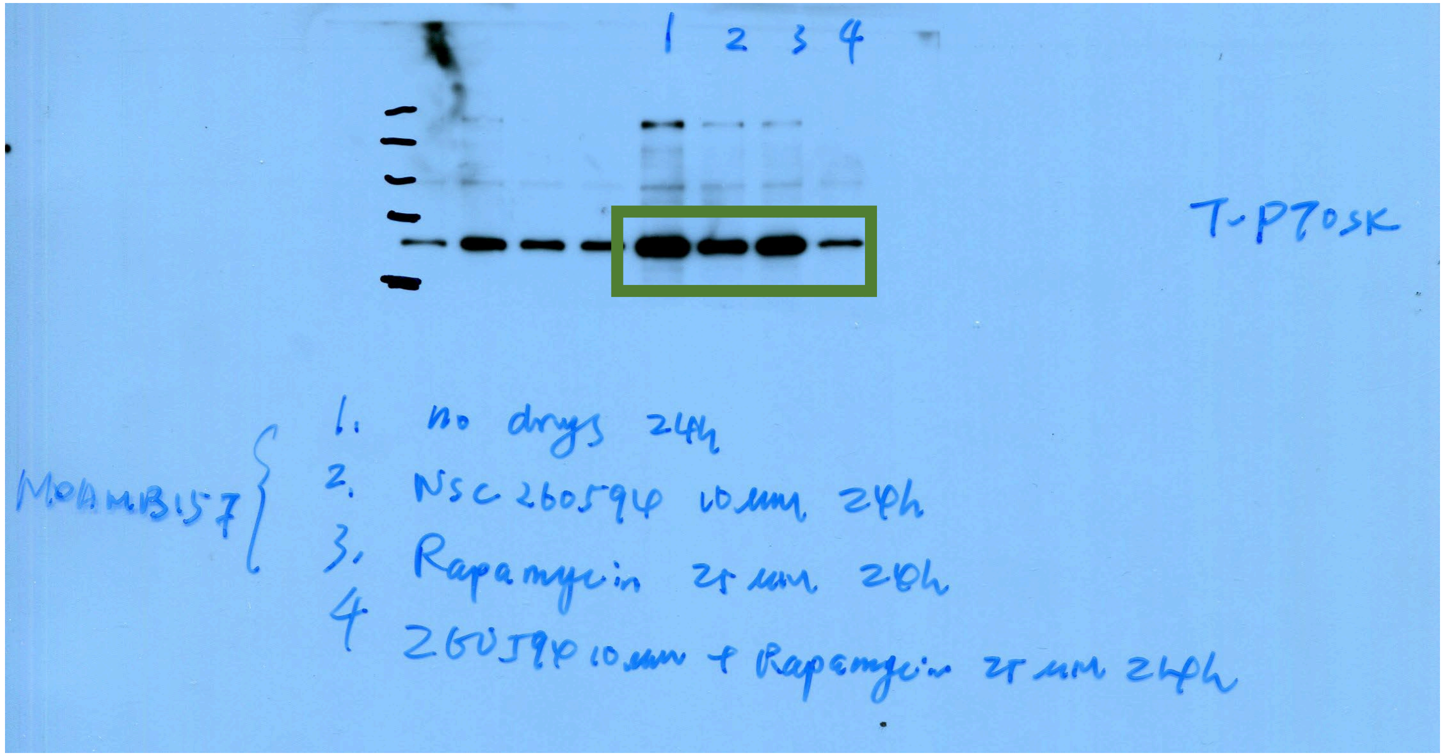

Figure 6D T-P70SK- lanes 1,2,3,4

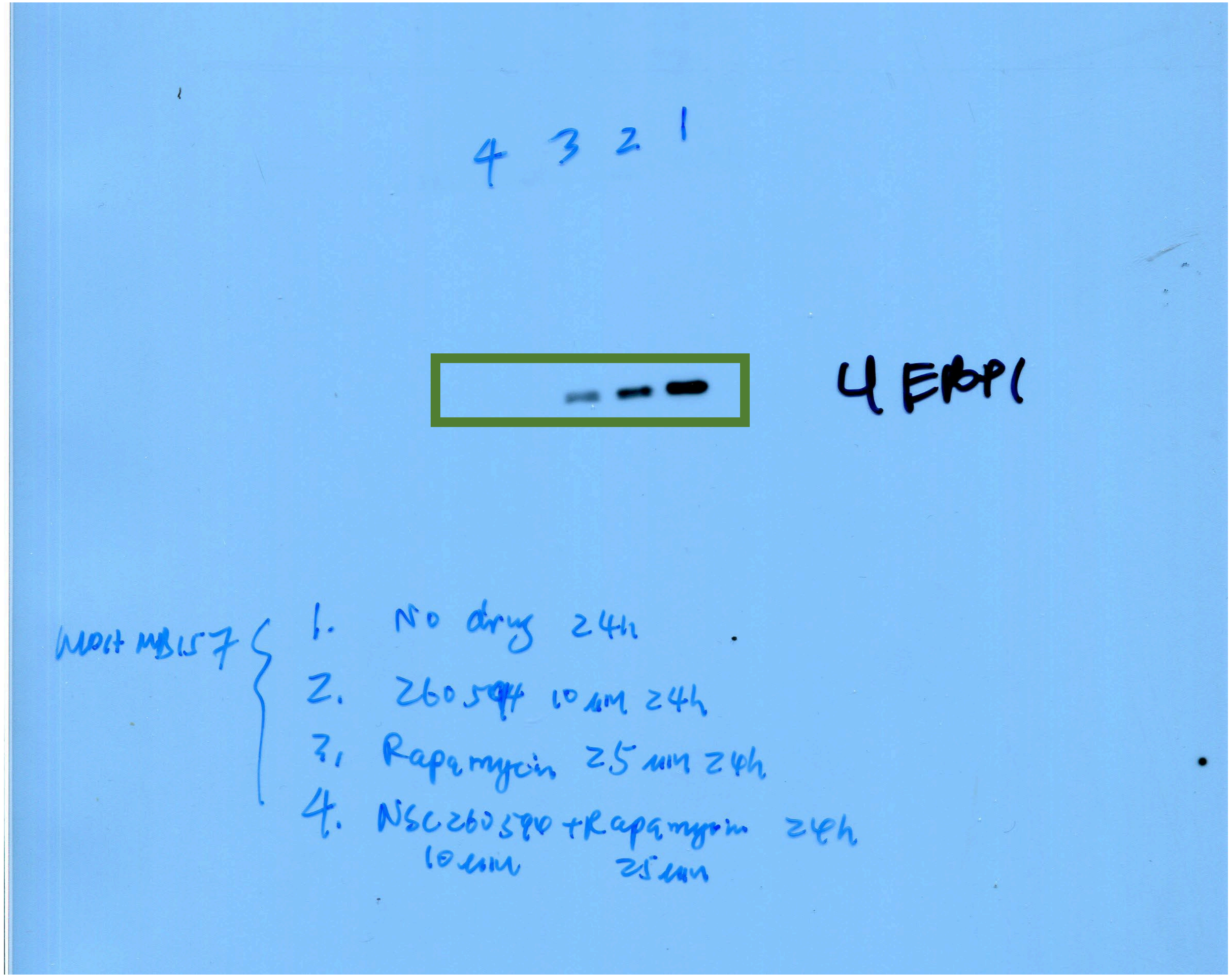

Figure 6D- p-4EBP1- lanes 1,2,3,4

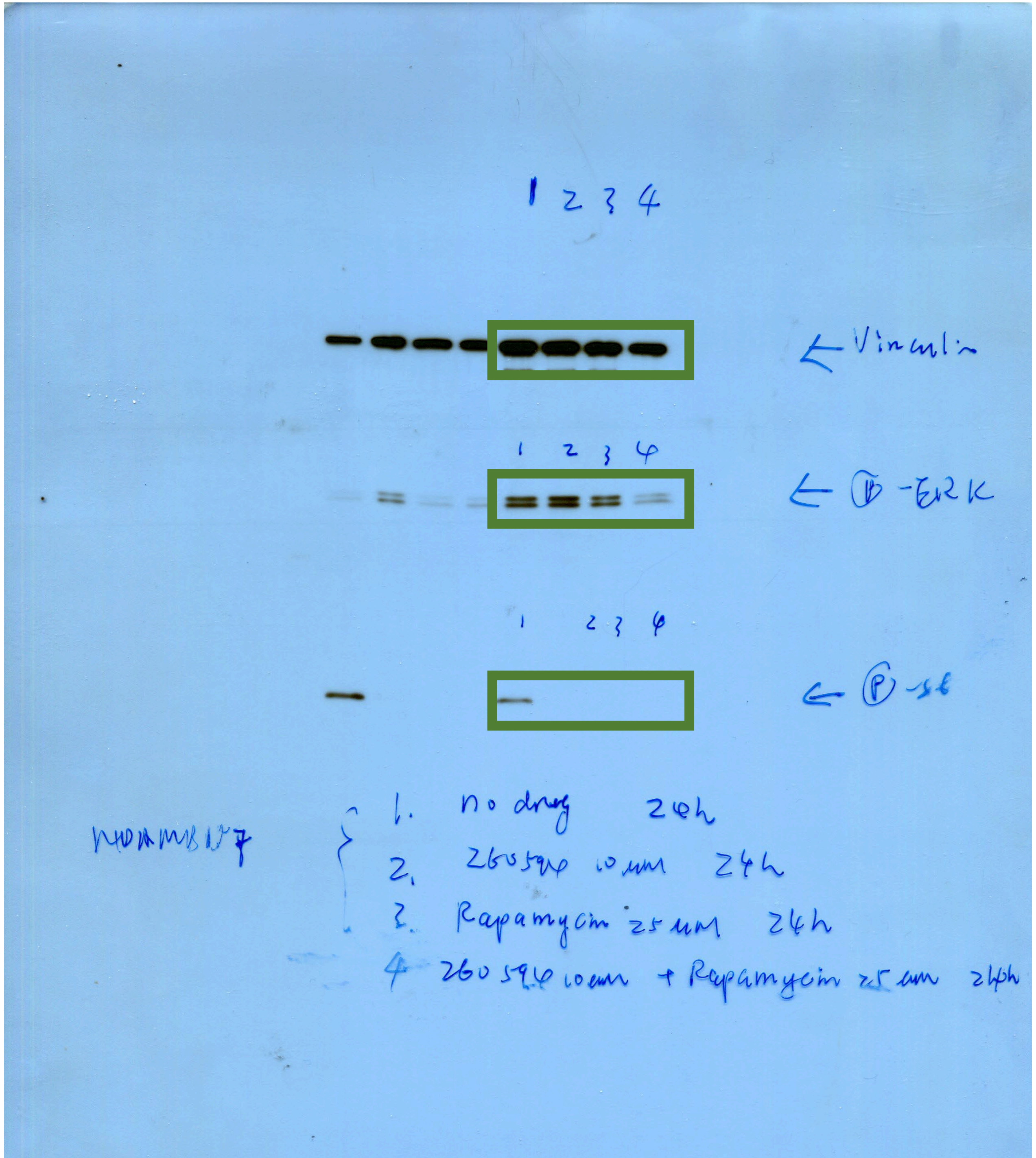

Figure 6D- vinculin- lanes 1,2,3,4

Figure 6D- p-ERK- lanes 1,2,3,4

Figure 6D- p-S6- lanes 1,2,3,4

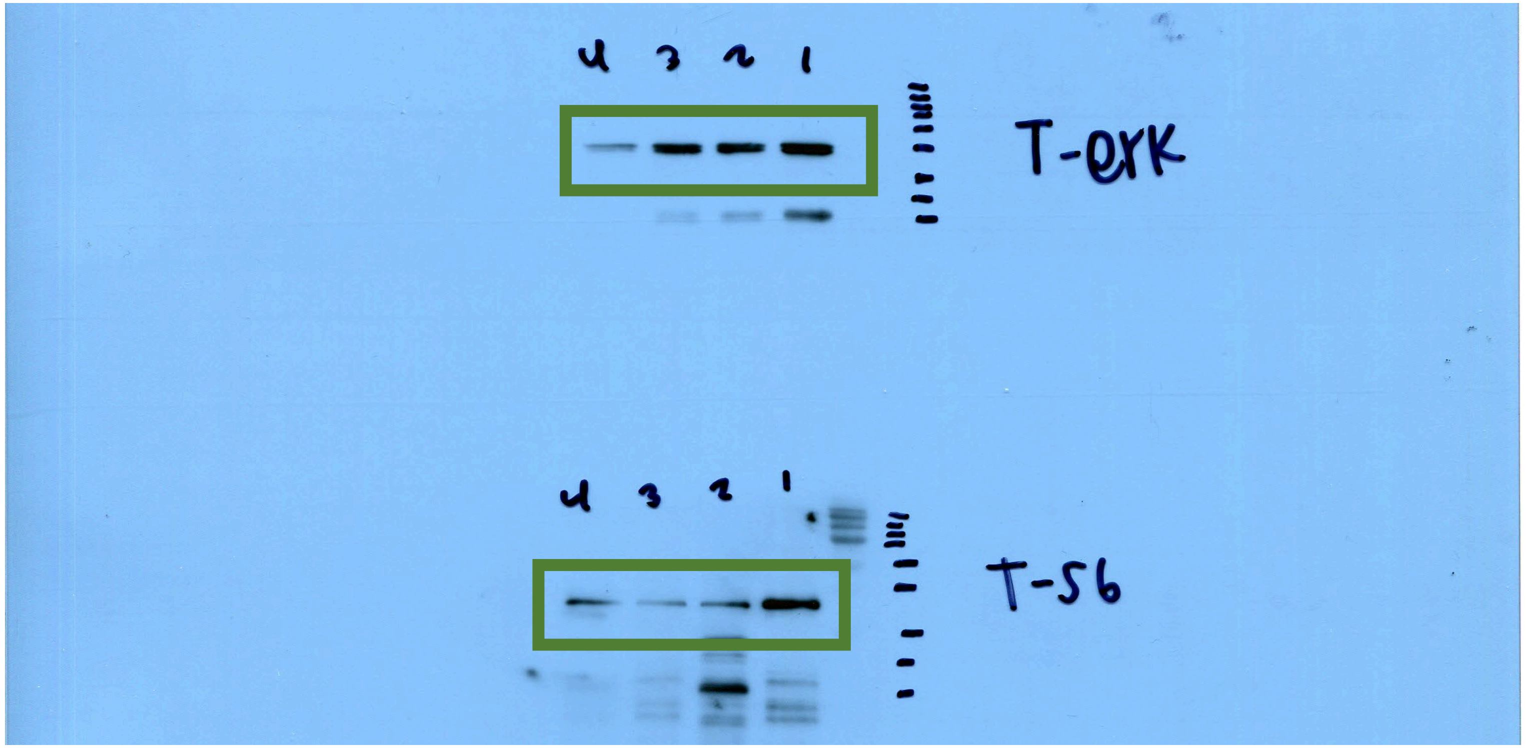

Figure 6D- T-ERK- lanes 1,2,3,4

Figure 6D- T-S6- lanes 1,2,3,4
